# Cognitive impairment and transcriptomic profile in hippocampus of young mice after multiple neonatal exposures to sevoflurane

**DOI:** 10.18632/aging.102326

**Published:** 2019-10-03

**Authors:** Shao-Yong Song, Xiao-Wen Meng, ZhengYuan Xia, Hong Liu, Juan Zhang, Qing-Cai Chen, Hua-Yue Liu, Fu-Hai Ji, Ke Peng

**Affiliations:** 1 Department of Anesthesiology, First Affiliated Hospital of Soochow University, Suzhou, Jiangsu, China; 2 Department of Anesthesiology, University of Hong Kong, Hong Kong, China; 3 Department of Anesthesiology and Pain Medicine, University of California Davis Health System, Sacramento, CA 95817, USA

**Keywords:** sevoflurane, cognitive function, hippocampus, RNA sequencing, differentially expressed genes

## Abstract

Children with repeated inhalational anesthesia may develop cognitive disorders. This study aimed to investigate the transcriptome-wide response of hippocampus in young mice that had been exposed to multiple sevoflurane in the neonatal period. Mice received 3% sevoflurane for 2 h on postnatal day (PND) 6, 8, and 10, followed by arterial blood gas test on PND 10, behavioral experiments on PND 31–36, and RNA sequencing (RNA-seq) of hippocampus on PND 37. Functional annotation and protein-protein interaction analyses of differentially expressed genes (DEGs) and quantitative reverse transcription polymerase chain reaction (qPCR) were performed. Neonatal sevoflurane exposures induced cognitive and social behavior disorders in young mice. RNA-seq identified a total of 314 DEGs. Several enriched biological processes (ion channels, brain development, learning, and memory) and signaling pathways (oxytocin signaling pathway and glutamatergic, cholinergic, and GABAergic synapses) were highlighted. As hub-proteins, Pten was involved in nervous system development, synapse assembly, learning, memory, and behaviors, Nos3 and Pik3cd in oxytocin signaling pathway, and Cdk16 in exocytosis and phosphorylation. Some top DEGs were validated by qPCR. This study revealed a transcriptome-wide profile in mice hippocampus after multiple neonatal exposures to sevoflurane, promoting better understanding of underlying mechanisms and investigation of preventive strategies.

## INTRODUCTION

In a recent epidemiologic study of 20,922 children in the US, approximately 1 in 7 children experienced at least 1 episode of surgical procedure under general anesthesia before 3 years of age [1]. Of them, more than 1 in 4 children who received general anesthesia were at high risk for neurodevelopmental problems, as defined by Food and Drug Administration warning—“repeated or lengthy use of general anesthetic and sedative agents may affect brain development in children < 3 years” [1, 2].

Over the years, studies have shown that anesthesia and surgery during childhood was associated not only with postoperative behavioral changes, but also with long-term deficits in neurocognitive function, learning ability, and academic performance [3–7]. Recent studies provided evidence that a single brief anesthesia for approximately 1 h in infancy or under age 3 did not have significant impact on neurodevelopment [8–10]; however, in children receiving multiple anesthesia exposures, their parents reported problems of behaviors, executive function, and reading [11]. Animal studies also indicated neurotoxicity after neonatal exposure to general anesthesia [12–17]. To date, the extent of the risk of neurotoxicity associated with anesthetics and the exact mechanisms remain elusive.

Sevoflurane is the most popular inhalational anesthetic agent for pediatric patients, with excellent properties of respiratory tolerance, rapid onset, rapid offset, and hemodynamic stability [18]. Recent studies showed that repeated sevoflurane exposures in the neonatal period led to subsequent learning impairment, memory deficits, and behavioral abnormalities in the young animal [12, 19–21]. However, these studies investigated the expression of several genes, related proteins, and a small number of signaling pathways.

Several brain regions including hippocampus, parahippocampal cortex, entorhinal cortex, retrosplenial cortex, and dorsolateral prefrontal cortex are involved in the cognitive processes [22, 23]. Of these, the hippocampus plays an essential role in navigation, cognition, and memory. Animal studies showed that gene knockout or pharmacological intervention in the hippocampus caused loss of learning ability and memory storage [24, 25]. In addition, the hippocampus and prefrontal cortex are related to impairments in cognitive function and memory during aging [26, 27].

In this study, the model of multiple neonatal exposures to sevoflurane was used to assess cognitive function, social interaction, anxiety, depression, and stereotyped behaviors in young mice. The main objective was to reveal the genome-wide expression profile and differentially expressed genes (DEGs) in the hippocampus between sevoflurane-treated and control mice by using the high-throughput mRNA-sequencing (RNA-seq). The biological processes, signaling pathways, and protein interactions of target DEGs were analyzed. Furthermore, real-time quantitative reverse transcription polymerase chain reaction (qPCR) assays were performed to validate the RNA-seq results.

## RESULTS

### Multiple neonatal exposures to sevoflurane led to cognitive and social behavior impairment in the young mice

**Figure 1:**
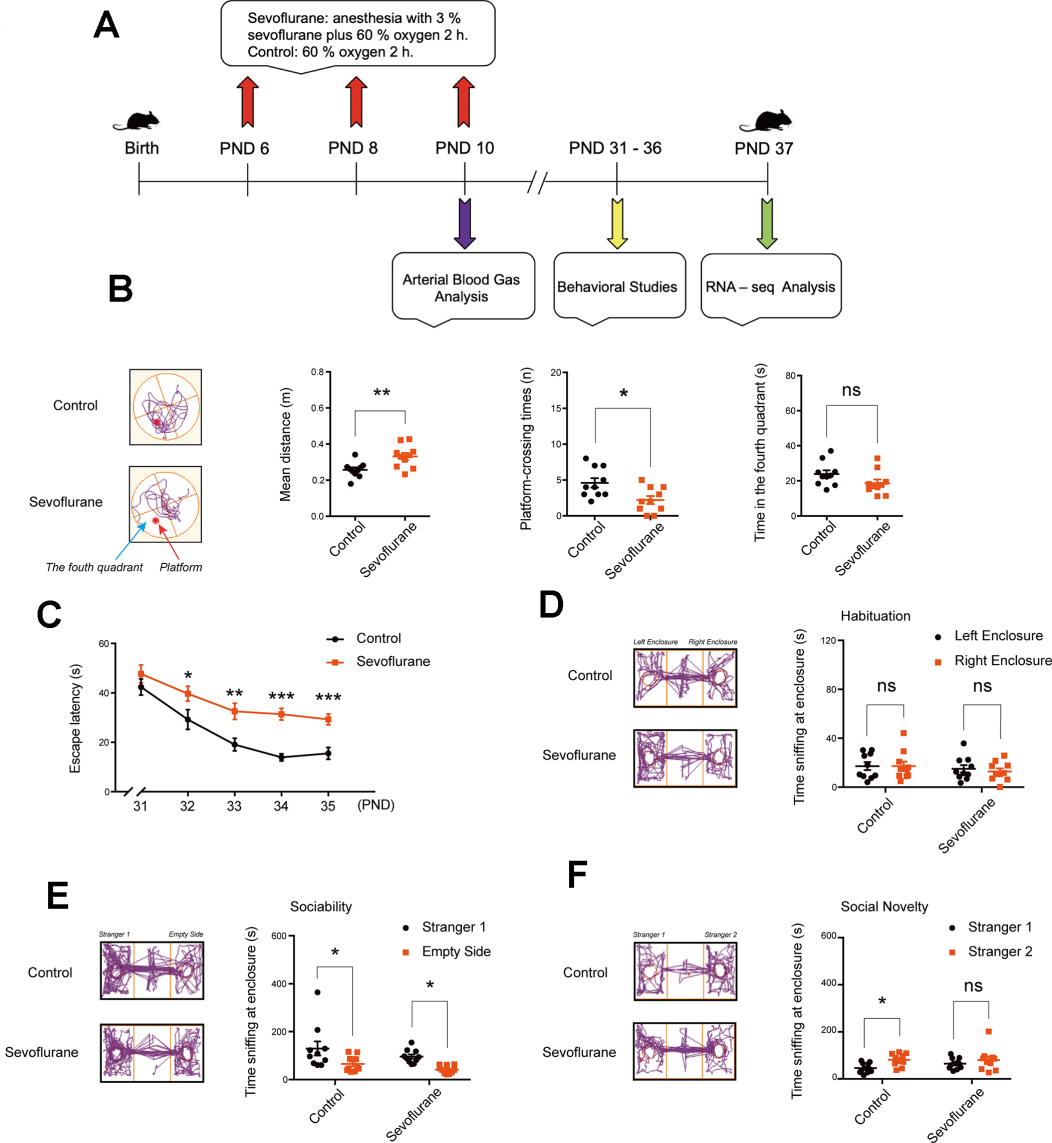
**Cognitive function and social behaviors in young mice after multiple sevoflurane exposures during the neonatal period.** (**A**) 3% sevoflurane 2 h daily on postnatal day (PND) 6, 8, and 10, arterial blood test on PND 10, behavioral test on PND 31–36, and RNA-seq analysis of hippocampal tissues on PND 37. (**B**) Morris water maze (testing phase on PND 36) showing longer mean distance from the original platform area and fewer platform-crossing times in the sevoflurane group, without significant difference in time spent in the fourth quadrant. (**C**) Morris water maze (training phase on PND 31–35) showing longer escape latency in the sevoflurane group. (**D**) Social interaction test on habituation showing no significant difference in time of sniffing at the left and right empty enclosures for both groups. (**E**) Social interaction test on sociability showing that both groups spent more time sniffing at Stranger 1 than at the empty enclosure. (**F**) Social interaction test on preference for social novelty showing that the control group, but not sevoflurane group, spent more time sniffing at Stranger 2 than Stranger 1. n = 10. *p < 0.05, **p < 0.01, ***p < 0.001 for the comparisons shown.

The study design is presented in Figure 1A. After sevoflurane anesthesia on postnatal day (PND) 6, 8, and 10, arterial blood gas tests were performed on PND 10, behavioral tests were carried out on PND 31–36, and the hippocampal tissues were harvested on PND 37 for RNA-Seq analysis.

Cognitive function was evaluated using the Morris water maze (MWM) test. In the testing phase on PND 36, mice in the sevoflurane group had longer mean distance from the platform (0.33 ± 0.02 vs. 0.26 ± 0.01 m, *p* = 0.006) and fewer platform-crossing times (2.20 ± 0.55 vs. 4.60 ± 0.65, *p* = 0.011) compared to the control group (Figure 1B), while time spent in the fourth quadrant did not differ between the two groups. In the training phase on PND 32–35, the sevoflurane group showed longer escape latency than the control group (31.36 ± 2.37 vs. 13.91 ± 1.44 s, *p* < 0.0001 for PND 34; Figure 1C).

In addition, social interaction tests were carried out. In the habituation session, both groups spent similar time sniffing at the left and right empty enclosures (Figure 1D). In the sociability session, both groups spent more time sniffing at Stranger 1 than at the empty enclosure (Figure 1E). In the preference for social novelty session, the control group spent more time sniffing at Stranger 2 than Stranger 1 (81.05 ± 6.60 vs. 42.31 ± 5.64 s, *p* = 0.02; Figure 1F), while the sevoflurane group showed no difference (80.48 ± 15.53 vs. 64.73 ± 7.57 s, *p* = 0.48).

### Multiple neonatal exposures to sevoflurane did not induce anxiety-like, depression-like, or stereotyped behaviors

First, in the open field test, two groups had similar time of stay in the center, speed of movement, and distance traveled (Supplementary Figure 1A–1C), indicating that sevoflurane did not induce anxiety-like behaviors. Next, in the elevated plus maze and light-dark box tests, the two groups had similar time in the open arms, numbers of open and closed arms entries, and time in the caliginous section (Supplementary Figure 1D–1G), suggesting no significant depression-like behaviors. Finally, in the self-grooming test, similar numbers and time of self-grooming showed that sevoflurane did not induce stereotyped behaviors (Supplementary Figure 1H–1I).

### Oxygenation and homeostasis were maintained during sevoflurane anesthesia

To rule out the possibility that the specific changes noted in behaviors or gene expression may be due to poor oxygenation, arterial blood samples were taken for analysis at PND 10 in the sevoflurane group. The value of *P*O_2_ was lower at 115 min than that at 5 min (89.25 ± 1.66 vs. 94.13 ± 0.91 mmHg, *p* = 0.02; Supplementary Figure 2A), but both within the normal range. In addition, the other parameters including *P*CO_2_, Hct, pH, Na^+^, K^+^, Ca^+^, and Cl^-^ remained stable (Supplementary Figure 2B–2H).

### RNA-seq analysis identified 49 up-regulated and 265 down-regulated DEGs

**Figure 2:**
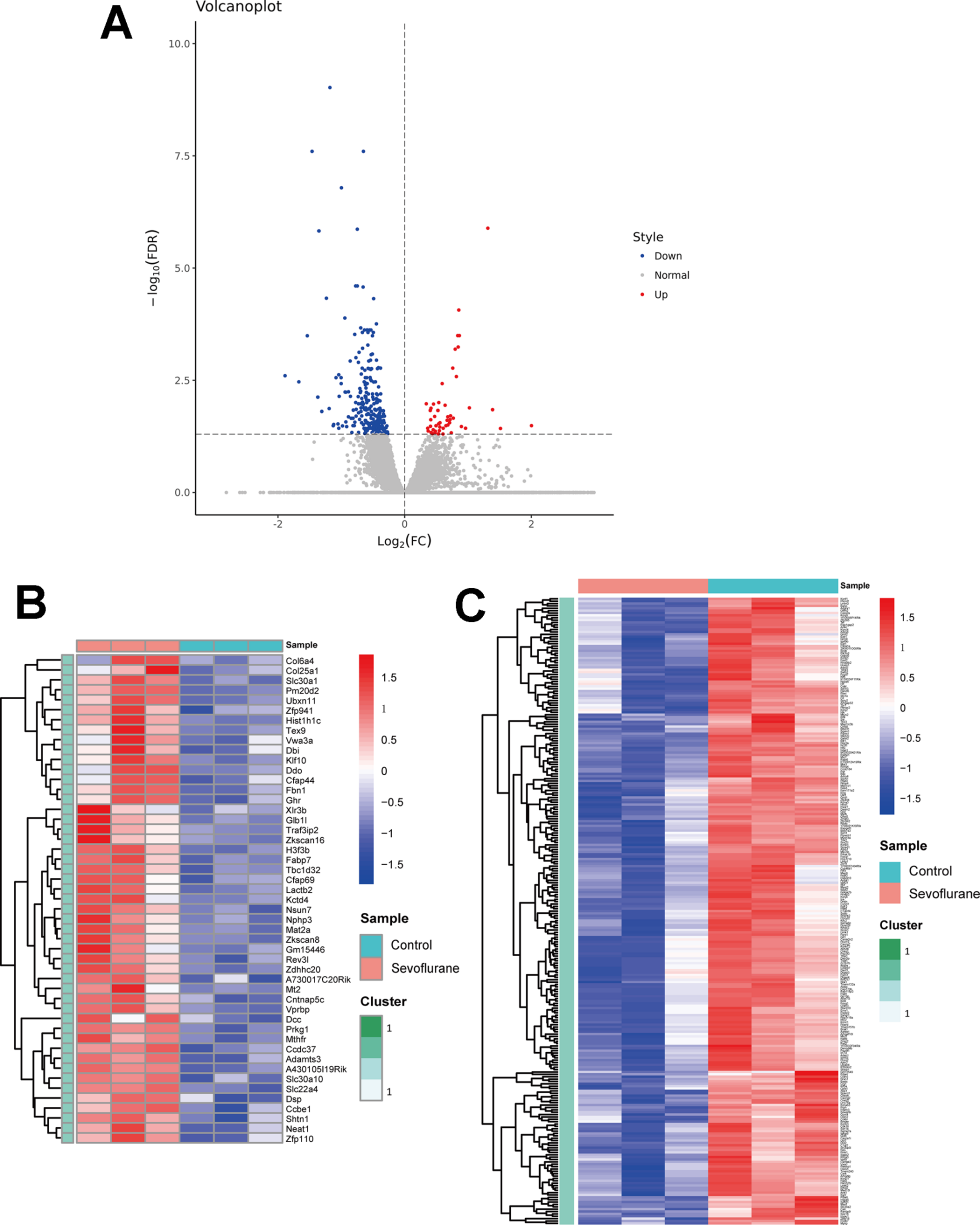
**Volcano plot and heatmaps of DEGs in hippocampal tissues between sevoflurane-treated and control mice.** (**A**) Volcano plot showing up-regulated (red dots) and down-regulated (blue dots) DEGs and normally expressed genes (gray dots). (**B**) Heatmap of 49 up-regulated DEGs. (**C**) Heatmap of 265 down-regulated DEGs.

RNA-seq analysis showed the expression level of 17,107 genes in the mice hippocampus of the sevoflurane and control groups. Of these, a total of 314 DEGs were determined (Figure 2A), including 49 up-regulated genes (Figure 2B, Supplementary Table 2) and 265 down-regulated genes (Figure 2C, Supplementary Table 3). These DEGs present differential expression profiles, which distinguishes the sevoflurane group from the control group apparently. The top 20 up- and down-regulated DEGs are listed in Table 1.

**Table 1:** Top 20 up-regulated and down-regulated DEGs.

**Gene**	**log_2_FC**	***p*-value**	**FDR**	**Location**
**Up-regulated**				
*Hist1h1c*	1.312676	5.32E-10	1.29E-06	chr13:23738807-23739531
*Fabp7*	0.853611	9.19E-08	8.57E-05	chr10:57784923-57788450
*Neat1*	0.836954	6.96E-07	0.000319	chr19:5824710-5845478
*Tbc1d32*	0.862511	7.10E-07	0.000319	chr10:56014294-56228689
*Klf10*	0.843942	1.46E-06	0.000573	chr15:38294406-38300711
*Mt2*	0.796616	1.74E-06	0.00064	chr8:94172618-94173567
*Fbn1*	0.758222	6.25E-06	0.001693	chr2:125300594-125506438
*Ccbe1*	0.815541	1.23E-05	0.002613	chr18:66056856-66291838
*Prkg1*	0.591146	2.20E-05	0.003748	chr19:30564487-31765033
*Pm20d2*	0.537705	9.04E-05	0.009937	chr4:33170406-33189737
*H3f3b*	0.34208	0.000102	0.01057	chr11:116021961-116024504
*Slc30a1*	0.456209	0.000105	0.010728	chr1:191906781-191913247
*Cntnap5c*	0.637167	0.000114	0.011397	chr17:57769570-58410342
*Ddo*	1.020954	0.000134	0.01302	chr10:40630011-40649931
*Mthfr*	0.416183	0.000141	0.013283	chr4:148041189-148059562
*Cfap44*	1.387494	0.000160	0.014272	chr16:44394799-44482428
*Mat2a*	0.40471	0.000174	0.014828	chr6:72432799-72439558
*Zkscan8*	0.524755	0.000175	0.014828	chr13:21513221-21531114
*Cfap69*	0.72325	0.000263	0.019481	chr5:5580982-5664232
*Slc30a10*	0.474247	0.000284	0.020126	chr1:185454848-185468761
**Down-regulated**				
*Sdf2l1*	-1.1809	7.82E-14	9.48E-10	chr16:17130138-17132383
*E130012A19Rik*	-1.4612	5.89E-12	2.51E-08	chr11:97627387-97629716
*Gatsl2*	-0.65162	6.22E-12	2.51E-08	chr5:134099748-134141758
*Rims4*	-0.99919	5.35E-11	1.62E-07	chr2:163863881-163918683
*Doc2a*	-0.74835	6.73E-10	1.36E-06	chr7:126847553-126852705
*Klf4*	-1.35382	8.59E-10	1.49E-06	chr4:55527137-55532475
*Efhd2*	-0.74812	1.74E-08	2.51E-05	chr4:141858142-141874920
*Ccdc184*	-0.7742	1.86E-08	2.51E-05	chr15:98167806-98170134
*Stx1a*	-0.65659	2.18E-08	2.65E-05	chr5:135023572-135051099
*Sox18*	-1.23674	4.24E-08	4.68E-05	chr2:181669837-181671640
*Nr2f1*	-0.49017	4.73E-08	4.78E-05	chr13:78188973-78198982
*Cit*	-0.94487	1.49E-07	0.000129	chr5:115845656-116006341
*9430020K01Rik*	-0.44648	2.16E-07	0.000175	chr18:4634929-4682869
*Kcnab3*	-0.69399	2.83E-07	0.000215	chr11:69326258-69333041
*Lasp1*	-0.63517	3.35E-07	0.000239	chr11:97799672-97838764
*Cacna1i*	-0.53254	3.60E-07	0.000239	chr15:80287238-80398292
*Fosl2*	-0.59154	3.75E-07	0.000239	chr5:32136472-32157839
*Pdia6*	-0.55648	3.98E-07	0.000239	chr12:17266595-17284770
*Xbp1*	-0.59461	4.14E-07	0.000239	chr11:5520641-5525993
*Pgr*	-0.5794	5.15E-07	0.000271	chr9:8899833-8968611

DEG, differentially expressed gene; FDR, false discovery rate.

### Gene Ontology (GO) analysis revealed significantly enriched GO terms and interactions of biological processes

**Figure 3:**
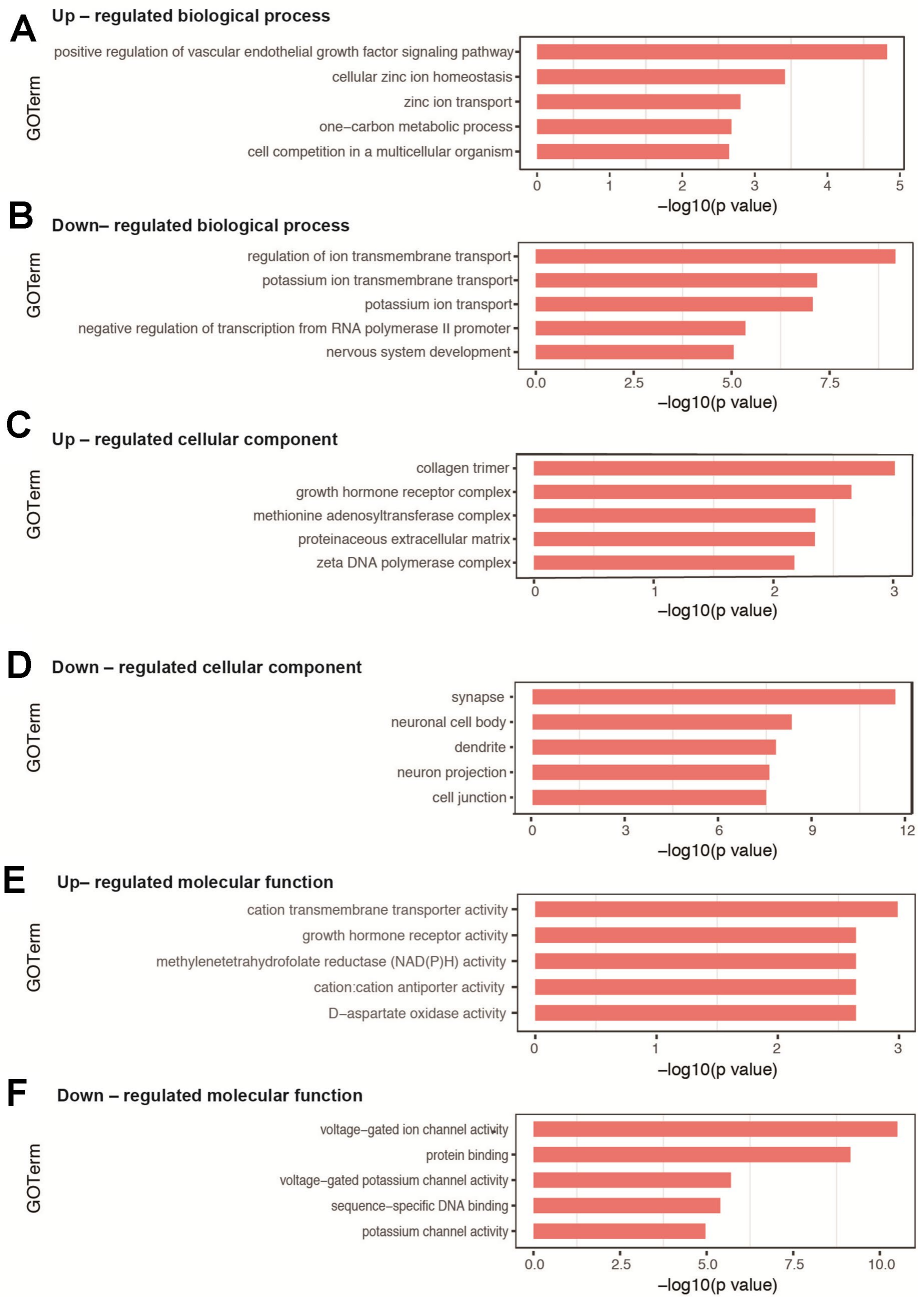
**GO analysis of DEGs in hippocampal tissues between sevoflurane-treated and control mice.** (**A**, **B**) Top 5 GO terms of biological process based on up- and down-regulated DEGs. (**C**, **D**) Top 5 GO terms of cellular component based on up- and down-regulated DEGs. (**E**, **F**) Top 5 GO terms of molecular function based on up- and down-regulated DEGs. Blue columns indicate terms without significant difference.

For the biological process, the top 10 categories with associated DEGs are presented in Table 2. The top 3 enriched GO terms of up-regulated DEGs were positive regulation of vascular endothelial growth factor signaling pathway, cellular zinc ion homeostasis, and zinc ion transport (Figure 3A), while those associated with down-regulated DEGs included regulation of ion transmembrane transport, potassium ion transmembrane transport, and potassium ion transport (Figure 3B). Several significantly enriched biological processes were related to brain development (nervous system development, exocytosis, synapse assembly, and phosphorylation) and cognitive function (learning, memory, and long-term memory), all of which were associated with down-regulated DEGs. All GO terms of biological process are listed in Supplementary Tables 4–5.

**Table 2:** Top 10 enriched BP terms of up-regulated and down-regulated DEGs.

**ID**	**Term**	**DEG(s)**	***p*-value**	**FDR**	**Enrichment**
**Up-regulated**					
GO:1900748	positive regulation of vascular endothelial growth factor signaling pathway	*Adamts3, Ccbe1*	1.503E-05	0.0040579	294.52778
GO:0006882	cellular zinc ion homeostasis	*Slc30a1, Mt2*	0.0003851	0.0406079	67.967949
GO:0006829	zinc ion transport	*Slc30a1, Slc30a10*	0.0015749	0.0406079	33.983974
GO:0006730	one-carbon metabolic process	*Mat2a, Mthfr*	0.0020959	0.0406079	29.452778
GO:0035212	cell competition in a multicellular organism	*Vprbp*	0.0022635	0.0406079	441.79167
GO:0090230	regulation of centromere complex assembly	*H3f3b*	0.0022635	0.0406079	441.79167
GO:0030575	nuclear body organization	*Neat1*	0.0022635	0.0406079	441.79167
GO:1990245	histone H2A-T120 phosphorylation	*Vprbp*	0.0022635	0.0406079	441.79167
GO:0036018	cellular response to erythropoietin	*Mt2*	0.0022635	0.0406079	441.79167
GO:0071584	negative regulation of zinc ion transmembrane import	*Slc30a1*	0.0022635	0.0406079	441.79167
**Down-regulated**					
GO:0034765	regulation of ion transmembrane transport	*Cacng2, Kcnh3, Kcna3, Kcnj4, Cacna1i, Kcnc4, Kcnab3, Kcnj11, Scn1b, Kcnj9, Kcnf1, Kcnb1, Cacng7, Kcnk3, Kcnc1, Kcnj2*	6.587E-10	8.642E-07	7.3469317
GO:0071805	potassium ion transmembrane transport	*Kcnh3, Kcna3, Kcnk12, Kcnj4, Kcnc4, Kcnab3, Kcnj11, Kcnf1, Kcnb1, Kcnk3, Kcnc1, Kcnj2*	6.591E-08	3.715E-05	7.5871199
GO:0006813	potassium ion transport	*Kcnh3, Kcna3, Kcnj4, Kcnc4, Kcnab3, Kcnj11, Kcnj9, Kcnf1, Kcnb1, Kcnk3, Kcnc1, Kcnj2*	8.495E-08	3.715E-05	7.4159818
GO:0000122	negative regulation of transcription from RNA polymerase II promoter	*Scrt1, Fgfr3, Mxi1, Egr1, Pou3f3, Zbtb16, Nfix, Bcl6, Nr2f1, Klf5, Tbx3, Eng, Xbp1, Fezf2, Satb2, Calr, Dab2ip, Ppard, Foxg1, Sox18, Hivep1, Dusp26, Klf4, Cry1*	4.431E-06	0.0014534	2.850652
GO:0007399	nervous system development	*Igsf9, Ina, Pou3f3, Sema7a, Nes, Nr2f1, Lmtk2, Fezf2, Cit, Ndel1, Brsk1, Nrsn1, Gdpd5, Sema6c, Bax, Pten*	8.858E-06	0.0023242	3.6734659
GO:0018105	peptidyl-serine phosphorylation	*Ulk1, Adrbk1, Pink1, Lmtk2, Brsk1, Ttbk1, Csnk1e, Sbk1*	1.945E-05	0.0041297	6.9952169
GO:0006811	ion transport	*Cacng2, Kcnh3, Kcna3, Slc38a2, Kcnk12, Kcnj4, Cacna1i, Lasp1, Kcnc4, Fxyd7, Kcnab3, Kcnj11, Grid1, Scn1b, Kcnj9, Kcnf1, Kcnb1, Cacng7, Kcnk3, Kcnc1, Kcnj2*	2.203E-05	0.0041297	2.8111885
GO:0006887	exocytosis	*Stxbp1, Doc2a, Cdk16, Stx1a, Rims3, Srcin1, Rims4, Cplx1*	3.524E-05	0.0057795	6.4465724
GO:0007416	synapse assembly	*Plxnd1, Farp1, Shank2, Clstn3, Pten*	4.606E-05	0.0067145	12.453606
GO:0016310	phosphorylation	*Camk2n2, Prkar1b, Fgfr3, Ulk1, Limk1, Adrbk1, Cdk16, Pink1, Lmtk2, Stk32c, Nlk, Cit, Brsk1, Pik3cd, Ttbk1, Pak6, Camkk2, Tnk2, Mast2, Csnk1e, Sbk1, Cerk*	5.219E-05	0.0068479	2.575874

BP: biological process; DEG: differentially expressed gene; FDR: false discovery rate.

The enriched GO terms of DEGs were also classified according to cellular component and molecular function. For cellular component, the most significantly categories were collagen trimer for up-regulated DEGs (Figure 3C) and synapse for down-regulated DEGs (Figure 3D). All GO terms on cellular component are shown in Supplementary Tables 6–7. For molecular function, the most significantly categories were cation transmembrane transporter activity for up-regulated DEGs (Figure 3E) and voltage-gated ion channel activity for down-regulated DEGs (Figure 3F). All GO terms on molecular function are shown in Supplementary Tables 8–9.

**Figure 4:**
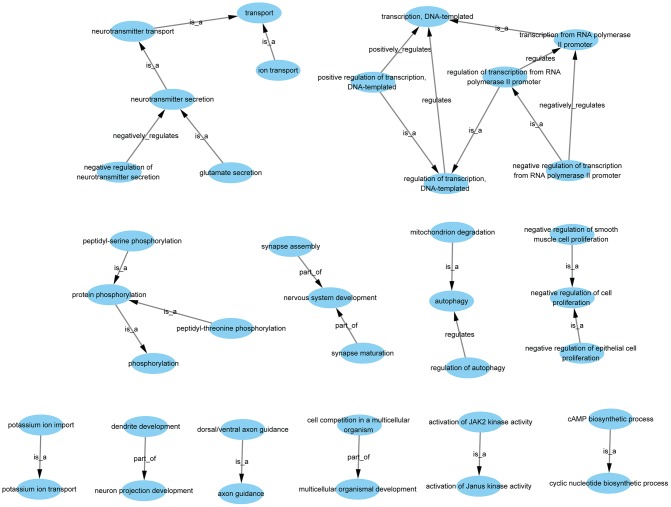
**GO-tree analysis based on biological process.** Arrow source, a GO term at a lower level; arrow target, a GO term at a higher level. Relation: is a, the downstream GO term is completely subordinate to the upstream GO term; part of, a part of the downstream GO term is subordinate to the upstream GO term; regulates, the downstream GO term can regulate the upstream GO term; positive regulates, the downstream GO term can regulate the upstream GO term positively; negative regulates, the downstream GO term can regulate the upstream GO term negatively.

GO-Tree analysis illustrated the interactions within the biological processes, as shown in Figure 4. A total of 37 GO terms were involved, with 5 terms associated with up-regulated genes and 32 terms associated with down-regulated genes (Supplementary Table 10). There were several types of relations including complete subordination, partly subordination, positive regulation, and negative regulation within the GO terms.

### Pathway analysis revealed significantly enriched signaling pathways and their relationship

**Figure 5:**
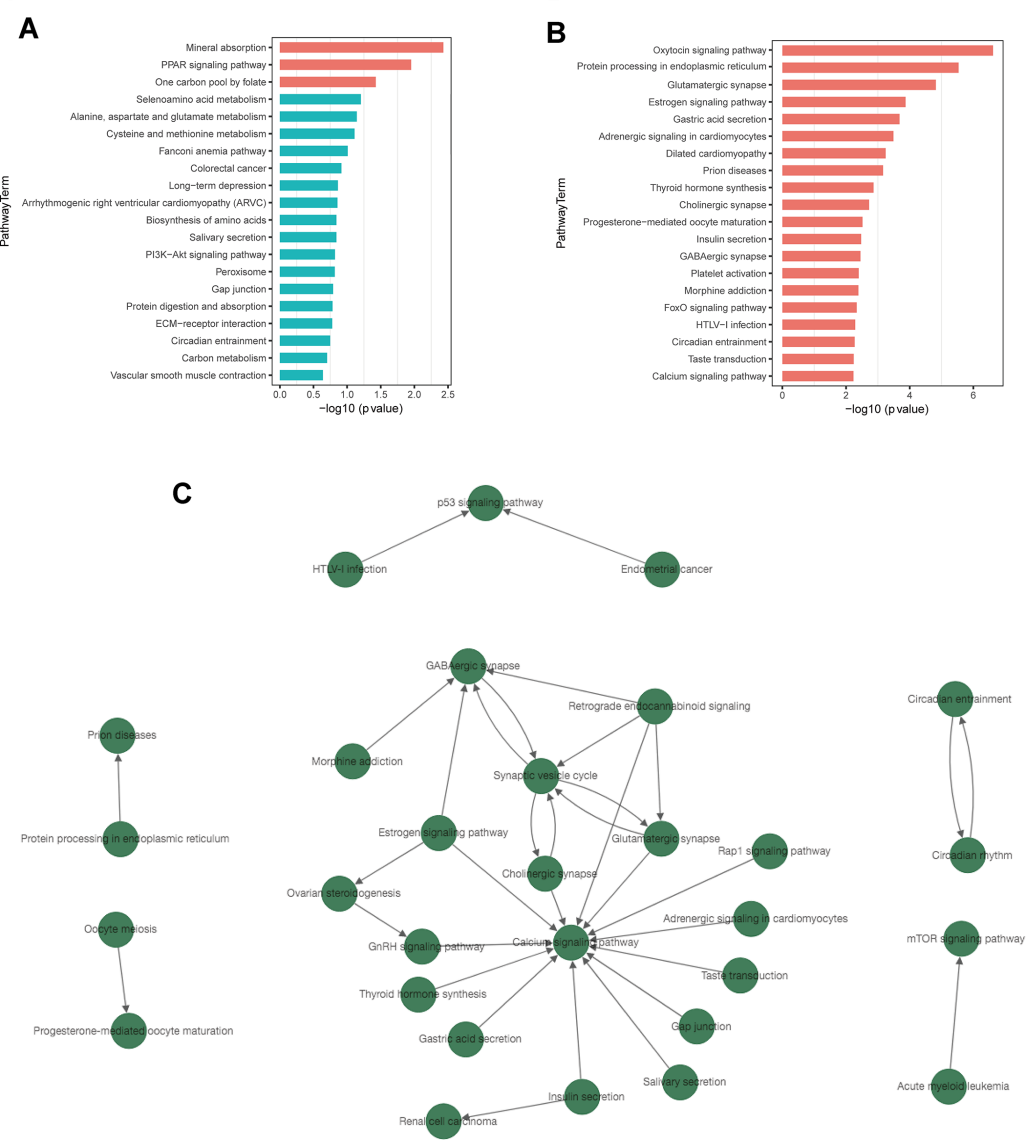
**KEGG pathway enrichment and pathway-tree analysis.** (**A**) Top 20 enriched pathways of up-regulated DEGs. (**B**) Top 20 enriched pathways of down-regulated DEGs. (**C**) Pathway-tree analysis showing relationship between pathway terms. Arrow source, an upstream signal pathway; arrow target, a downstream signaling pathway. Blue columns indicate pathways without significant difference.

Kyoto Encyclopedia of Genes and Genomes (KEGG) pathway analysis revealed 40 significantly enriched signaling pathways, including 3 associated with up-regulated DEGs and 37 associated with down-regulated DEGs. The top enriched pathways for up- and down-regulated genes are presented in Table 3. Mineral absorption, PPAR signaling pathway, and one carbon pool by folate were the top 3 enriched pathways associated with up-regulated DEGs (Figure 5A). For down-regulated DEGs, the top 3 enriched pathways were oxytocin signaling pathway, protein processing in endoplasmic reticulum, and glutamatergic synapse (Figure 5B). Several significantly enriched signaling pathways were associated with the regulation of synapses, including glutamatergic synapse, cholinergic synapse, and GABAergic synapse. All results of KEGG pathway enrichment are listed in Supplementary Tables 11–12.

**Table 3:** Signaling pathway enrichment of up-regulated and down-regulated DEGs.

**ID**	**Term**	**DEG(s)**	***p*-value**	**FDR**	**Enrichment**
**Up-regulated**					
PATH:04978	Mineral absorption	*Slc30a1, Mt2*	0.0037153	0.1263212	21.611594
PATH:03320	PPAR signaling pathway	*Dbi, Fabp7*	0.0111679	0.1898536	12.273251
PATH:00670	One carbon pool by folate	*Mthfr*	0.0375845	0.3333577	26.161404
**Down-regulated**					
PATH:04921	Oxytocin signaling pathway	*Cacng2, Adcy1, Kcnj4, Kcnj9, Adcy8, Pik3cd, Elk1, Adcy4, Camkk2, Cacng7, Nos3, Kcnj2*	2.428E-07	3.569E-05	6.5089481
PATH:04141	Protein processing in endoplasmic reticulum	*Ubqln4, Hspa5, Hyou1, Syvn1, Xbp1, Calr, Pdia4, Fbxo2, Pdia6, Bax, Man1c1*	2.924E-06	0.0002149	5.71341
PATH:04724	Glutamatergic synapse	*Slc38a2, Grm4, Adcy1, Grm2, Adrbk1, Glul, Adcy8, Adcy4, Shank2*	1.52E-05	0.000745	6.0733098
PATH:04915	Estrogen signaling pathway	*Adcy1, Kcnj9, Adcy8, Pik3cd, Fkbp4, Adcy4, Nos3*	0.0001353	0.0049722	6.1215107
PATH:04971	Gastric acid secretion	*Adcy1, Sst, Adcy8, Cckbr, Adcy4, Kcnj2*	0.0002082	0.0061217	6.9487418
PATH:04261	Adrenergic signaling in cardiomyocytes	*Cacng2, Adrb1, Adcy1, Scn1b, Adcy8, Pik3cd, Adcy4, Cacng7*	0.0003244	0.0079469	4.601404
PATH:05414	Dilated cardiomyopathy	*Cacng2, Adrb1, Adcy1, Adcy8, Adcy4, Cacng7*	0.0005682	0.0119312	5.7776056
PATH:05020	Prion diseases	*Egr1, Hspa5, Elk1, Bax*	0.0006871	0.0126259	9.7944171
PATH:04918	Thyroid hormone synthesis	*Adcy1, Hspa5, Adcy8, Pdia4, Adcy4*	0.0013702	0.0223796	6.0352922
PATH:04725	Cholinergic synapse	*Adcy1, Kcnj4, Adcy8, Pik3cd, Adcy4, Kcnj2*	0.0018898	0.0277797	4.591133

DEG: differentially expressed gene; FDR: false discovery rate.

Pathway network analysis showed the upstream and downstream relationship of the significantly enriched signaling pathways (Figure 5C). A total of 30 signaling pathways were involved, all of which were associated with down-regulated DEGs. (Supplementary Table 13). Calcium signaling pathway, synaptic vesicle cycle, and GABAergic synapse were the top 3 pathways with rich connections with other pathways.

### GO and KEGG pathway analysis of top 20 up- and down-regulated DEGs

To further investigate the role of top 20 up- and down-regulated DEGs, GO and KEGG analyses were carried out. These genes were labeled in the volcano plot (Supplementary Figure 3A), and their differential expression profiles were visualized in heatmaps (Supplementary Figure 3B–3C). Based on these DEGs, significantly enriched biological processes, cellular components, and molecular functions were categorized by GO analysis (Supplementary Figure 3D–3E). Besides, KEGG pathway analysis found several significantly enriched pathways associated with up- and down-regulated DEGs (Supplementary Figure 3F–3G).

### Protein-protein interaction (PPI) network revealed several hub proteins

**Figure 6:**
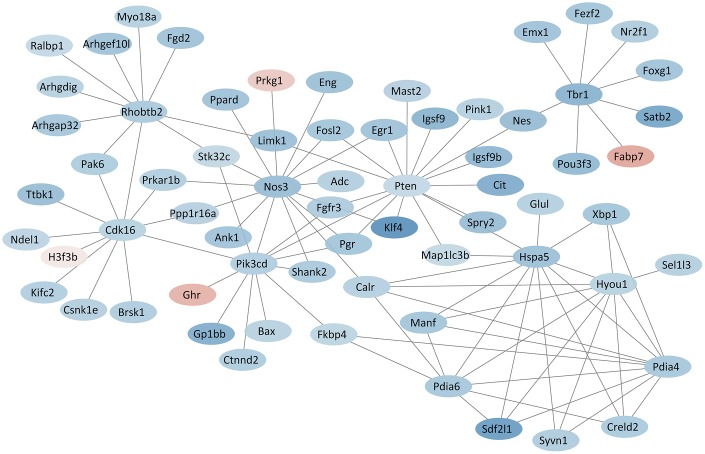
**PPI network based on hub proteins.** Red and blue colors indicate proteins with up- and down-regulated expression, respectively.

PPI network analysis showed the interactions among the proteins. Several hub proteins that had rich connectivity with other proteins were identified. Pten (degree = 16), Nos3 (degree = 14), Pik3cd (degree = 12), Cdk16 (degree = 11), Rhobtb2 (degree = 11), Hyou1 (degree = 9), Hspa5 (degree = 9), Pdia4 (degree = 8), Pdia6 (degree = 8), Tbr1 (degree = 8) were the top 10 hub proteins (Figure 6). All results of protein-protein interactions are shown in Supplementary Figure 4.

### RNA-Seq results were validated by qPCR

**Figure 7:**
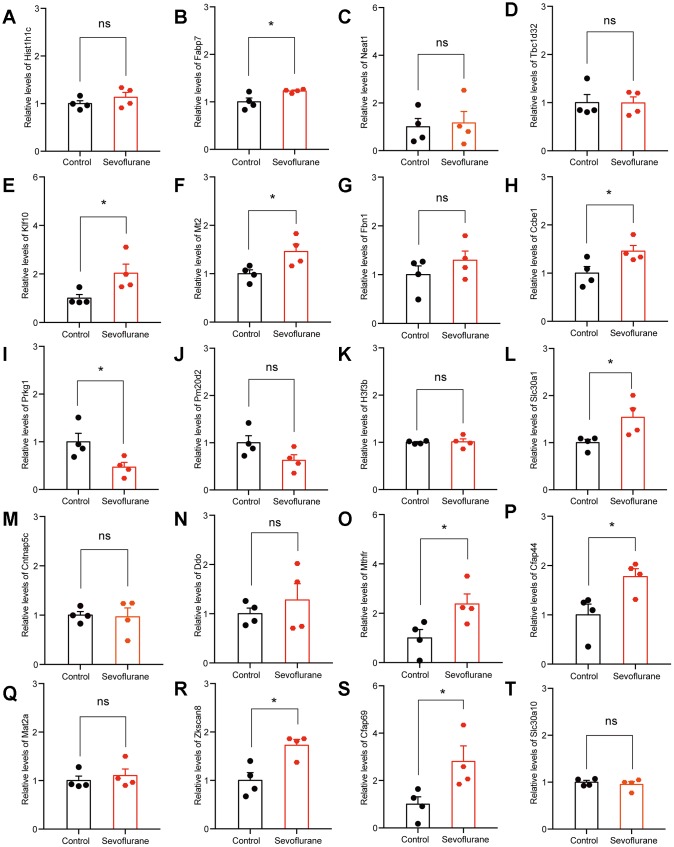
**qPCR verification of top 20 up-regulated DEGs.** (**A**) *Hist1h1c*. (**B**) *Fabp7*. (**C**) *Neat1*. (**D**) *Tbc1d32*. (**E**) *Klf10*. (**F**) *Mt2*. (**G**) *Fbn1*. (**H**) *Ccbe1*. (**I**) *Prkg1*. (**J**) *Pm20d2*. (**K**) *H3f3b*. (**L**) *Slc30a1*. (**M**) *Cntnap5c*. (**N**) *Ddo*. (**O**) *Mthfr*. (**P**) *Cfap44*. (**Q**) *Mat2a*. (**R**) *Zkscan8*. (**S**) *Cfap69*. (**T**) *Slc30a10*. n = 4. *p < 0.05 for the comparisons shown.

**Figure 8:**
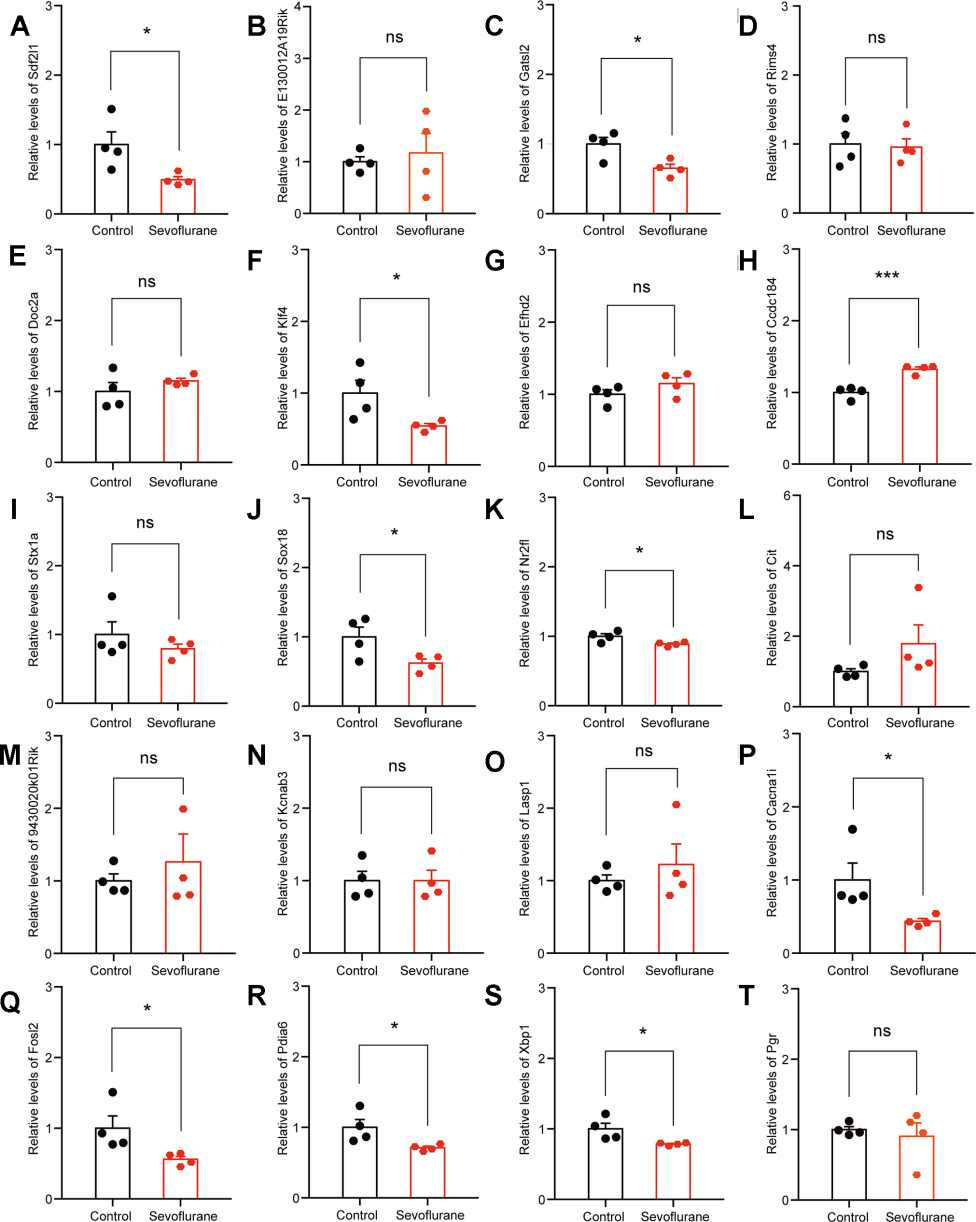
**qPCR verification of top 20 down-regulated DEGs.** (**A**) *Sdf2l1*. (**B**) *E130012A19Rik*. (**C**) *Gatsl2*. (**D**) *Rims4*. (**E**) *Doc2a*. (**F**) *Klf4*. (**G**) *Efhd2*. (**H**) *Ccdc184*. (**I**) *Stx1a*. (**J**) *Sox18*. (**K**) *Nr2f1*. (**L**) *Cit*. (**M**) *9430020K01Rik*. (**N**) *Kcnab3*. (**O**) *Lasp1*. (**P**) *Cacna1i*. (**Q**) *Fosl2*. (**R**) *Pdia6*. (**S**) *Xbp1*. (**T**) *Pgr*. n = 4. *p < 0.05, ***p < 0.001 for the comparisons shown.

The top 20 up- and down-regulated DEGs from RNA-seq analysis were verified by qPCR. For up-regulated DEGs, both RNA-seq analysis and qPCR revealed 9 genes with significantly positive expression change (*Fabp7*, *Klf10*, *Mt2*, *Ccbe1*, *Slc30a1*, *Mthfr*, *Cfap44*, *Zkscan8*, and *Cfap69*), while the expression of *Prkg1* showed a negative change in the qPCR results (Figure 7). For down-regulated DEGs, both methods identified 9 genes with a significantly negative expression change (*Sdf2l1*, *Gatsl2*, *Klf4*, *Sox18*, *Nr2fl*, *Cacna1i*, *Fols2*, *Pdia6*, and *Xbp1*), while qPCR showed that the expression of *Ccdc184* changed positively (Figure 8).

## DISCUSSION

This is the first study revealing the genome-wide response of the transcriptome of the hippocampus of young mice to neonatal sevoflurane exposures for 3 non-consecutive days. Sevoflurane induced cognitive impairment and social behavior disorders in young mice, but not anxiety-like, depression-like, or stereotyped behaviors. During the sevoflurane procedures, oxygenation and homeostasis were maintained. RNA-seq analysis of the hippocampal tissues identified a total of 314 DEGs and revealed their functional enrichment and interactions. The most significantly enriched biological processes included positive regulation of vascular endothelial growth factor signaling pathway and regulation of ion transmembrane transport. The most significantly enriched signaling pathways included mineral absorption and oxytocin signaling pathway. Pten, Nos3, and Pik3cd were the top 3 hub proteins. The expression of some top DEGs were confirmed by qPCR.

During the human brain development, a unique peak of synaptogenesis occurs in the primary sensorimotor cortex near the birth, temporal cortex at 9 months, and prefrontal cortex at age 3, which is also known as a window of vulnerability [28]. For rodents, the vulnerability window seems to happen on PND 7–30 when exposure to sevoflurane affected brain development [14, 29, 30]. In terms of the sevoflurane concentration, our study used a clinically relevant anesthetic concentration (minimum alveolar concentration of 3.3 ± 0.2% in human neonates) [31]. In addition, studies suggest that 3% sevoflurane did not significantly alter the values of pH, arterial oxygen tension, arterial carbon dioxide tension, or electrolyte contents in mice [31, 32], which was in line with our blood gas results. Thus, the anesthesia protocol of 3% sevoflurane for 2 h on PND 6, 8, and 10 was used in this study. Regarding the MWM test, mice at one-month age may experience relative higher stress in the Morris water maze as compared to adult mice. In fact, MWM performance may be affected by several factors including training procedure, apparatus, and characteristics of the experimental animals (species, strain, sex, age, nutritional status, stress, and infection) [33]. The MWM test has been successfully used to determine cognitive function in young mice at 1-month age in recent studies [12, 20].

Studies showed that neonatal sevoflurane exposures induced long-term neurodegenerative change, learning disability, and memory impairment [12, 34–37]. In the immature brain, sevoflurane caused excitatory effects of the type A gamma-amino butyric acid (GABA-A) receptors, which might contribute to sevoflurane-induced long-term cognitive disorders [38, 39]. Besides, the function of N-Methyl-d-aspartate (NMDA) receptors which is critical for learning and memory has been interfered by repeated sevoflurane anesthesia at the neonatal stage [40, 41]. On the other hand, sevoflurane-induced neurotoxicity was alleviated by treatments of tanshinone IIA, dexmedetomidine, or JNK inhibitor [42–44]. In this study, MWM and social interaction tests confirmed that multiple exposures to sevoflurane in the neonatal period induced cognitive impairment in the young mice. Moreover, RNA-seq analysis of the hippocampus revealed several genes and biological processes related to brain development, including nervous system development (GO:0007399; *Igsf9, Ina, Pou3f3, Sema7a, Nes, Nr2f1, Lmtk2, Fezf2, Cit, Ndel1, Brsk1, Nrsn1, Gdpd5, Sema6c, Bax, Pten*), exocytosis (GO:0006887; *Stxbp1, Doc2a, Cdk16, Stx1a, Rims3, Srcin1, Rims4, Cplx1*), synapse assembly (GO:0007416; *Plxnd1, Farp1, Shank2, Clstn3, Pten*), and phosphorylation (GO:0016310; *Camk2n2, Prkar1b, Fgfr3, Ulk1, Limk1, Adrbk1, Cdk16, Pink1, Lmtk2, Stk32c, Nlk, Cit, Brsk1, Pik3cd, Ttbk1, Pak6, Camkk2, Tnk2, Mast2, Csnk1e, Sbk1, Cerk*). In addition, there were several biological processes related to learning and memory, including learning (GO:0007612; *Grm4*, *Ctnnd2*, *Pak6*, *Shank2*), memory (GO:0007613; *Adrb1*, *Pak6*, *Shank2*, *Pten*), learning or memory (GO:0007611; *Prkar1b*, *Egr1*, *Pten*), and long-term memory (GO:0007616; *Adcy1*, *Adcy8*). Of note, all the above-mentioned genes were significantly down-regulated in the hippocampus of sevoflurane-treated mice, suggesting potential new mechanisms which underlie the cognitive impairment by sevoflurane exposure.

In the GO analysis, several top enriched biological processes were related to ion channels. Cellular zinc ion homeostasis (GO:0006882; *Adamts3, Ccbe1*), zinc ion transport (GO:0006829; *Slc30a1, Slc30a10*), and negative regulation of zinc ion transmembrane import (GO:0071584; *Slc30a1*) were associated with up-regulated DEGs. Regulation of ion transmembrane transport (GO:0034765; *Cacng2, Kcnh3, Kcna3, Kcnj4, Cacna1i, Kcnc4, Kcnab3, Kcnj11, Scn1b, Kcnj9, Kcnf1, Kcnb1, Cacng7, Kcnk3, Kcnc1, Kcnj2*), potassium ion transmembrane transport (GO:0071805; *Kcnh3, Kcna3, Kcnk12, Kcnj4, Kcnc4, Kcnab3, Kcnj11, Kcnf1, Kcnb1, Kcnk3, Kcnc1, Kcnj2*), potassium ion transport (GO:0006813; *Kcnh3, Kcna3, Kcnj4, Kcnc4, Kcnab3, Kcnj11, Kcnj9, Kcnf1, Kcnb1, Kcnk3, Kcnc1, Kcnj2*), and ion transport (GO:0006811; *Cacng2, Kcnh3, Kcna3, Slc38a2, Kcnk12, Kcnj4, Cacna1i, Lasp1, Kcnc4, Fxyd7, Kcnab3, Kcnj11, Grid1, Scn1b, Kcnj9, Kcnf1, Kcnb1, Cacng7, Kcnk3, Kcnc1, Kcnj2*) were based on down-regulated DEGs. Consistently, previous findings support that sevoflurane could influence the activities of ion channels [45–48].

Cellular response to hypoxia and apoptotic process are also involved in the effects of sevoflurane on the neonatal brain. We found enriched biological process of cellular response to hypoxia associated with down-regulated DEGs (GO:0071456; *Egr1, Pink1, Ppard, Kcnk3*). Previous studies showed that early exposure to sevoflurane could cause apoptosis in the cerebral cortex and hippocampus [49, 50]. In this study, the GO analysis revealed a number of biological processes related to apoptosis, such as regulation of neuron apoptotic process (GO:0043523; *Grm2, Pink1, Bax*), positive regulation of apoptotic process (GO:0043065; *Adrb1, Zbtb16, Bcl6, Dab2ip, Zmat3, Nos3, Bax, Pten*), positive regulation of neuron apoptotic process (GO:0043525; *Fgfr3, Egr1, Bax*), and negative regulation of neuron apoptotic process (GO:0043524; *Stxbp1, Nes, Pink1, Bax, Sncb*). These results suggest that multiple sevoflurane exposures may affect neurodevelopment through regulating cellular response to hypoxia and the apoptotic process.

Autism spectrum disorder (ASD), a common childhood neurodevelopmental disorder, is characterized by social interaction impairment and repetitive behaviors [51]. It is unclear whether sevoflurane exposure may contribute to the development of ASD. Some studies reported that neonatal exposures to sevoflurane induced abnormal social behaviors [31, 52, 53], while others found no autism-like behaviors after sevoflurane anesthesia [36, 54]. In this study, multiple neonatal exposures to sevoflurane had a negative impact on social interactions, evidence by the loss of ability to distinguish a new mouse. This result can also be explained by the impairments of the frontal cortex functions, which is most vulnerable in the observed period of ontogenesis. A recent study found that multiple sevoflurane anesthesia impaired the prefrontal cortex functions in the developmental brain [55]. Our RNA-seq analysis identified 3 down-regulated genes (*Grid1*, *Shank2*, *Pten*) related to social behaviors, 5 down-regulated genes (*Sobp*, *Fezf2*, *Pak6*, *Apba1*, *Pten*) related to locomotory behaviors, and 1 up-regulated gene (*Ddo*) related to grooming-like stereotyped behaviors. The current results may help to detect the key genes for ASD treatment in further investigations.

Among the significantly enriched signaling pathways from KEGG analysis, oxytocin signaling pathway (PATH:04921; *Cacng2, Adcy1, Kcnj4, Kcnj9, Adcy8, Pik3cd, Elk1, Adcy4, Camkk2, Cacng7, Nos3, Kcnj2*) may play an important role in the sevoflurane-induced cognitive impairment. Tara et al. found that oxytocin signaling pathway in the hippocampus was essential for social recognition [56]. In this study, a significantly decreased activity of oxytocin signaling in the hippocampus was noted. In addition, several enriched signaling pathways of down-regulated DEGs were on the regulation of synapses, including glutamatergic synapse (PATH:04724; *Slc38a2, Grm4, Adcy1, Grm2, Adrbk1, Glul, Adcy8, Adcy4, Shank2*), cholinergic synapse (PATH:04725; *Adcy1, Kcnj4, Adcy8, Pik3cd, Adcy4, Kcnj2*), and GABAergic synapse (PATH:04727; *Slc38a2, Adcy1, Glul, Adcy8, Adcy4*). The above-mentioned pathways and related genes may be useful for further research.

The PPI network revealed several hub-proteins encoded by DEGs, including phosphatase and tensin homolog (Pten), nitric oxide synthase 3 (Nos3), phosphatidylinositol-4,5-bisphosphate 3-kinase catalytic subunit delta (Pik3cd), cyclin-dependent kinase 16 (Cdk16), Rho-related BTB domain containing 2 (Rhobtb2), hypoxia up-regulated 1 (Hyou1), heat shock protein 5 (Hspa5), protein disulfide isomerase associated 4 (Pdia4), protein disulfide isomerase associated 6 (Pdia6), and T-box brain gene 1 (Tbr1). Among these, Pten was involved in the processes of nervous system development, synapse assembly, learning and memory, and locomotory and social behaviors in the GO analysis. A recent study found that sevoflurane exposure on gestational day 14 induced neurotoxicity in the fetal brain of rats through the Pten signaling [57]. For other hub-proteins, Nos3 and Pik3cd were involved in the oxytocin signaling pathway in the KEGG analysis, and Cdk16 was involved in exocytosis and phosphorylation. Recent studies also showed that Nos3 and Pik3cd play an important role in neurodevelopmental disorders [58, 59].

This study has several limitations. First, the RNA-seq only tested the expression profile of mRNAs, without other types of RNAs such as lncRNAs or miRNAs. Second, the sample size of RNA-seq analysis was relatively small. Third, there were variations in gene expression detected by RNA-seq and qPCR, which may be due to the methodological or statistical differences. Fourth, the transcriptomic profile may need to be further validated by using the western blot experiment. Last, the entire hippocampal tissues of mice were harvested for RNA-seq. The discrepancy of gene expression and signaling pathway enrichment among different hippocampal regions including cornu amonis (CA)1, CA2, CA3, and gyrus dentatus should be considered. Therefore, based on the current findings, further experiments with larger sample size are needed to identify the role of key genes in response to multiple neonatal sevoflurane exposures.

In summary, multiple neonatal exposures to sevoflurane induced cognitive impairment and social behavior disorders in young mice. RNA-seq analysis identified 314 DEGs that encode proteins participating in relevant biological processes (ion channels, brain development, synapse assembly, learning, and memory) and signaling pathways (oxytocin signaling pathway, glutamatergic synapse, cholinergic synapse, and GABAergic synapse), via which the mice hippocampus responded to sevoflurane. With a novel insight into the sevoflurane-related neurotoxicity in developing brain, this study helps to provide a fundamental work for elucidation of underlying mechanisms and exploration of potential prophylactic targets.

## MATERIALS AND METHODS

### Animals and sevoflurane anesthesia

The experimental protocol was approved (protocol number: 201808A076) by the Institutional Animal Care and Use Committee of Soochow University (Suzhou, China). C57BL/6J mice were purchased from the Slaccas Laboratory (Shanghai, China) and received standard rodent food and water. The male pups were used in this study. Using a computer-generated table, the neonatal mice were randomly assigned to either of the two study groups (control or sevoflurane).

The animal model was described in a recent study [12]. On PND 6, 8, and 10, the sevoflurane group received 3% sevoflurane with 60% oxygen (balanced with nitrogen) for 2 h (2 L/min fresh gas for 3 min, followed by 1 L/min) in a chamber using the Datex-Ohmeda anesthesia system (Madison, WI, USA), while the control group received 60% oxygen in nitrogen for 2 h. The sevoflurane concentration was monitored and adjusted by using a gas analyzer (Vamos; Dräger Medical, Germany). The rectal temperature of mice was maintained at 37 ± 0.5 °C. After treatment, the mice were returned to home cages under standard care.

### Arterial blood gas analysis

Blood samples (100 μL each) were taken from the abdominal aorta at 5 min and 115 min during sevoflurane exposure on PND 10 (n = 8 for each group). The samples were then immediately analyzed by using a blood gas analyzer (Radiometer, ABL80 FLEX, Carlsbad, CA, USA). The values of pH, partial pressures of oxygen (*P*O_2_) and carbon dioxide (*P*CO_2_), hematocrit (Hct), Na^+^, K^+^, Ca^2+^, and Cl^-^ were recorded.

### Behavioral studies

The behavioral tests (n = 10 for each group) in this study included MWM at 03:00-5:00 pm on PND 31–36, social interaction test at 09:00-12:00 am on PND 31, open field test at 09:00-11:00 am on PND 32, elevated plus maze at 09:00-11:00 am on PND 33, light-dark box test at 09:00-11:00 am on PND 34, and self-grooming test at 09:00-11:00 am on PND 35. The motions of each mouse were monitored and recorded by the ANY-maze Behavior Tracking System (Stoelting Co., Wood Dale, IL, USA).

***MWM tests*** were conducted as previously described [60]. The water maze device was filled with opaque water using titanium dioxide to reach the level of 1.0 cm above the surface of a platform (diameter, 10 cm). Throughout the experiment, the water temperature was kept at 22 °C, and the surrounding environment remained quiet. In the training phase on PND 31–35, the mice were trained to reach the platform for 5 days with 4 trials per day, and the escape latency (time for mice to reach the platform) was recorded for the evaluation of spatial learning. In the testing phase on PND 36, the platform was removed, and mean distance from the original platform area, platform-crossing times, and time spent in the fourth quadrant (the platform quadrant) were recorded for the assessment of memory function. A heat lamp was used to warm and dry the mice before returning to home cages.

***Social interaction tests*** were performed as previously presented [61]. In the first session (habituation), the mouse was initially placed in the middle chamber, with free access to the left and right chambers. During 5 min of observation, the time of sniffing (direct snout-to-enclosure contact) at both enclosures in the center of chambers was recorded. In the second session (sociability), a different mouse, Stranger 1, was placed into one enclosure, and the motions of the experiment mouse were tracked for 10 min. The time of sniffing at Stranger 1 and at the empty enclosure were recorded. In the third session (preference for social novelty), another mouse, Stranger 2, was placed into the other enclosure, and the motions of the experiment mouse was tracked for 10 min. The time of sniffing at Stranger 1 and Stranger 2 was recorded.

***Open field tests*** were carried out to investigate the anxiety-like and exploratory behaviors, according to the previously described methods [62]. A central area of 10 × 10 cm^2^ was defined in the field box (40 × 40 × 40 cm^3^). The mouse was initially placed in the corner of the box and allowed to explore the field freely for 10 min. The time spent in the central area, speed of movement, and distance travelled were recorded.

***Elevated plus maze and light-dark box tests*** were used to assess the depression-like behaviors [63, 64]. The mouse was initially placed at the center of the maze and allowed to explore the maze freely for 10 min. The time of exploring open arms and number of open and closed arms entries were recorded. For the light-dark box, a large bright compartment was connected to a small caliginous compartment by a door of 7 cm. The mouse was initially placed in the center of the bright compartment and allowed to explore the two compartments freely for 5 min. The time spent in the caliginous compartment was recorded.

***Self-grooming tests*** were performed to evaluate the stereotyped behaviors using the previously described methods [65]. The mouse was housed individually and habituated to the circumstances in the open field box. Self-grooming behaviors (elliptical strokes, small strokes, bilateral strokes, flank licks, and tail and genital licks) [66], were tracked for 10 min. The numbers and time of self-grooming of each mouse were recorded.

### RNA isolation and sequencing

Total RNA was isolated from the mouse hippocampus using the Trizol reagent (Ambion, Shanghai, China). RNA purity and concentration were evaluated by using the Nanodrop ND-2000 spectrophotometer (Thermo Fisher Scientific, Waltham, MA, USA). RNA purity and integrity were checked by 1.5% agarose gel electrophoresis. RNA Integrity Number value > 7.0 was confirmed by using the Agilent 2100 Bioanalyzer (Agilent Technologies, Santa Clara, CA, USA).

Sequencing library was generated by using the TruSeq RNA preparation kit (Illumina, San Diego, CA, USA), following the manufacturer’s protocols. In Brief, the mRNA was purified by Oligo (dT) magnetic beads and fragmented into 200 bp short fragments. The complementary DNA (cDNA) was synthesized and purified. The concentration of cDNA was assessed by using the Qubit 2.0 (Thermo Fisher Scientific, Waltham, MA, USA), and the length of library fragments was determined by using the Agilent 2100 Bioanalyzer. Finally, sequencing was performed by using an Illumina HiSeq 2500 platform (Illumina, San Diego, CA, USA) and 125 bp paired-end reads were generated.

### Quality assessment of raw RNA-seq data

The raw data derived from RNA-seq was analyzed by using the FastQC (version 0.11.8, Babraham Bioinformatics, Babraham Institute, Cambridge, UK). To obtain the high-quality clean reads for subsequent analysis, the low-quality reads including the adaptor sequences, sequences with quality score of < 20, and reads with > 5% ambiguous bases (N bases) were removed from the sequencing data by using the Cutadapt v2.3 [67]. The raw data was uploaded onto the National Center for Biotechnology Information (NCBI), with the accession number of PRJNA556843 (available at: https://dataview.ncbi.nlm.nih.gov/object/PRJNA556843).

### Identification of DEGs

The clean reads from RNA-seq were aligned to mm10_UCSC genome by using the STAR tool [68]. Reads Per Kb per Million reads (RPKM) was used for the normalization and calculation of gene expression, and the DESeq2 algorithm (http://bioconductor.org/packages/DESeq2/) was applied to filter the DEGs [69]. The raw *p*-value was adjusted to false discovery rate (FDR), and FDR < 0.05 was considered as the cut-off criteria for DEGs screening [70]. To get an inclusive set of DEGs, we did not apply a fold change threshold for DEGs screening [71–74]. To overview the characteristics of gene expression profiles, volcano plots and heatmaps were generated by using the R package.

### Functional annotation of DEGs

GO analysis was performed to evaluate the functional enrichment of DEGs in the biological process, cellular component, and molecular function [75]. The annotations were downloaded from NCBI (www.ncbi.nlm.nih.gov/), UniProt (www.uniprot.org/), and Gene Ontology (www.geneontology.org/). A *p*-value < 0.05 was set as the threshold value. In addition, GO-Tree was constructed based on the significant GO-terms of DEGs. The GO-Tree is a directed acyclic graph, showing the relationships of GO-terms [76].

Kyoto Encyclopedia of Genes and Genomes (KEGG) pathway analysis was performed to analyze the enriched signaling pathways of DEGs based on the KEGG database (www.genome.jp/kegg/) [77]. A *p*-value < 0.05 was set as the threshold value. In addition to gene annotation in signaling pathways, the upstream and downstream relationships of the enriched pathways were shown by pathway network analysis.

### Protein-protein interaction network integration

The interactions among the proteins translated from the DEGs were analyzed by using the STRING database (version 10.5; www.string-db.org/), and the PPI network was visualized by using the Cytoscape software (www.cytoscape.org/) [78]. The proteins in the central node (hub proteins) may be the key proteins or genes that play an important role in regulating the physiological functions.

### qPCR

RNA was extracted from the mouse hippocampus (n = 4 for each group), and cDNA was synthesized using a reverse transcription kit (Transgen Biotech, Beijing, China). qPCR was carried out with the SYBR Green MasterMix in a 20 μL reaction volume by using the 7500 Fast Real-Time PCR System (Applied Biosystems, Bedford, MA, USA). Primers specific to the top 20 up- and down-regulated DEGs were synthesized by the Sangon Biotech (Shanghai, China), as shown in Supplementary Table 1. GAPDH was used as an internal control. Cycle threshold (Ct) value of a gene was obtained, and gene abundance was determined by using the 2^−ΔΔCt^ method.

### Statistical analysis

Data obtained from blood gas analysis, behavioral studies, and qPCR were checked for normal distribution and presented as mean ± standard error of the mean (SEM). Data were analyzed using the unpaired student’s *t* test or two-way analysis of variance (ANOVA) with repeated measurement followed by Bonferroni’s post hoc test as appropriate. All analyses were performed by using the GraphPad Prism 7.0 (San Diego, CA, USA). A two-tailed *p*-value < 0.05 indicates a statistically significant difference.

## Supplementary Material

Supplementary Figures

Supplementary Tables 1, 2, 10, 11 and 13

Supplementary Table 3

Supplementary Table 4

Supplementary Table 5

Supplementary Table 6

Supplementary Table 7

Supplementary Table 8

Supplementary Table 9

Supplementary Table 12
