# Supplementary material for: Cognitive impairment and transcriptomic profile in hippocampus of young mice after multiple neonatal exposures to sevoflurane
Source: Aging (Albany NY). 2019 Oct 3;11(19):8386–417. doi: 10.18632/aging.102326 (PMC6814607; doi:10.18632/aging.102326)
Supplement: Supplementary Figures [file aging-11-102326-s010.pdf]

SUPPLEMENTARY FIGURES

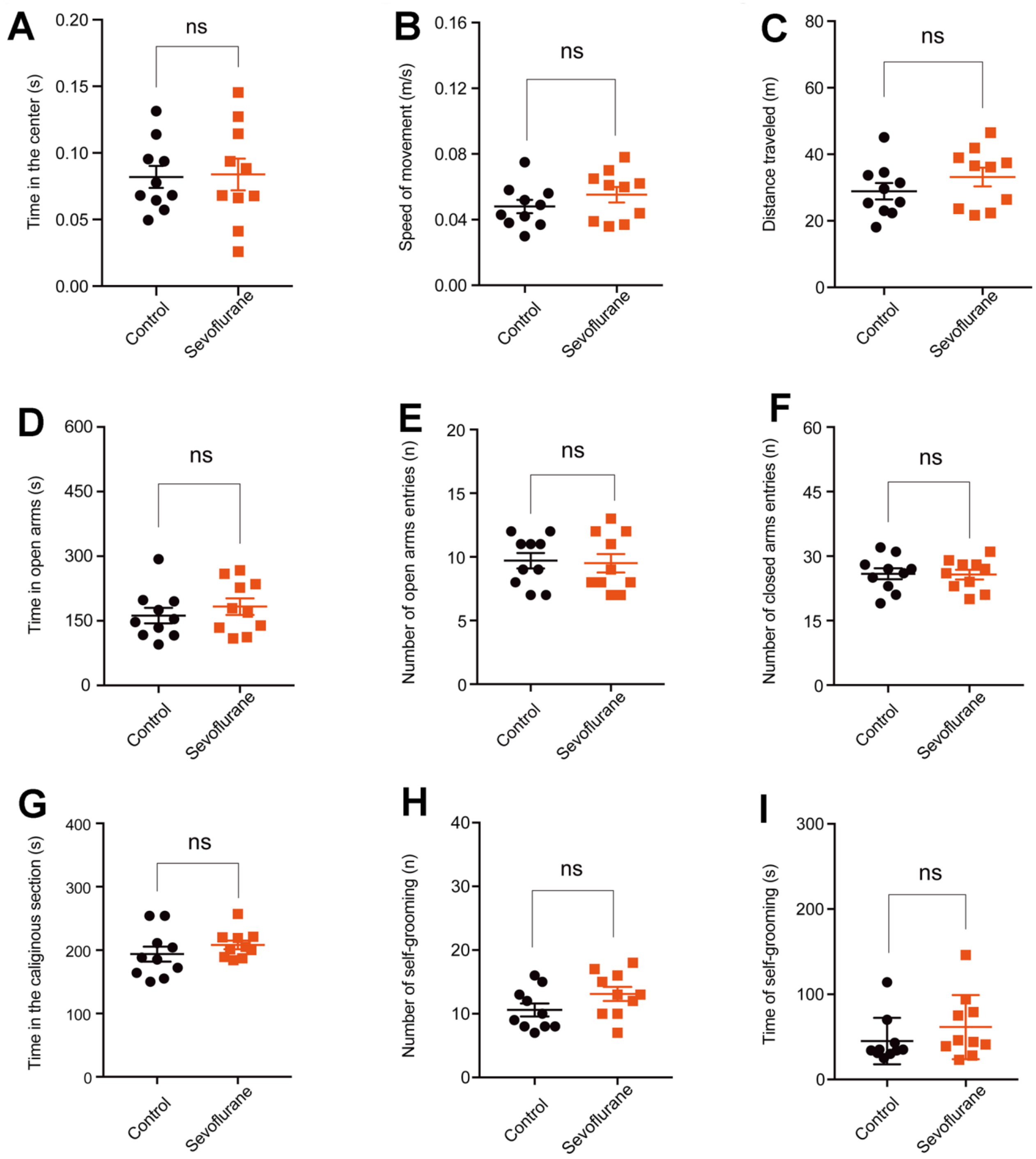

Supplementary Figure 1. Multiple sevoflurane exposures did not induce anxiety-like, depression-like, or stereotyped behaviors. (**A**–**C**) Open field test showing no significant differences in time spent in the center, speed of movement, or distance traveled. (**D**–**F**) Elevated plus maze test showing no significant differences in time of exploring open arms, numbers of open arms entries, or numbers of closed arms entries. (**G**) Light-dark box test showing no significant difference in time spent in the caliginous section. (**H**, **I**) Self-grooming test showing no significant differences in numbers and time of grooming. n = 10.

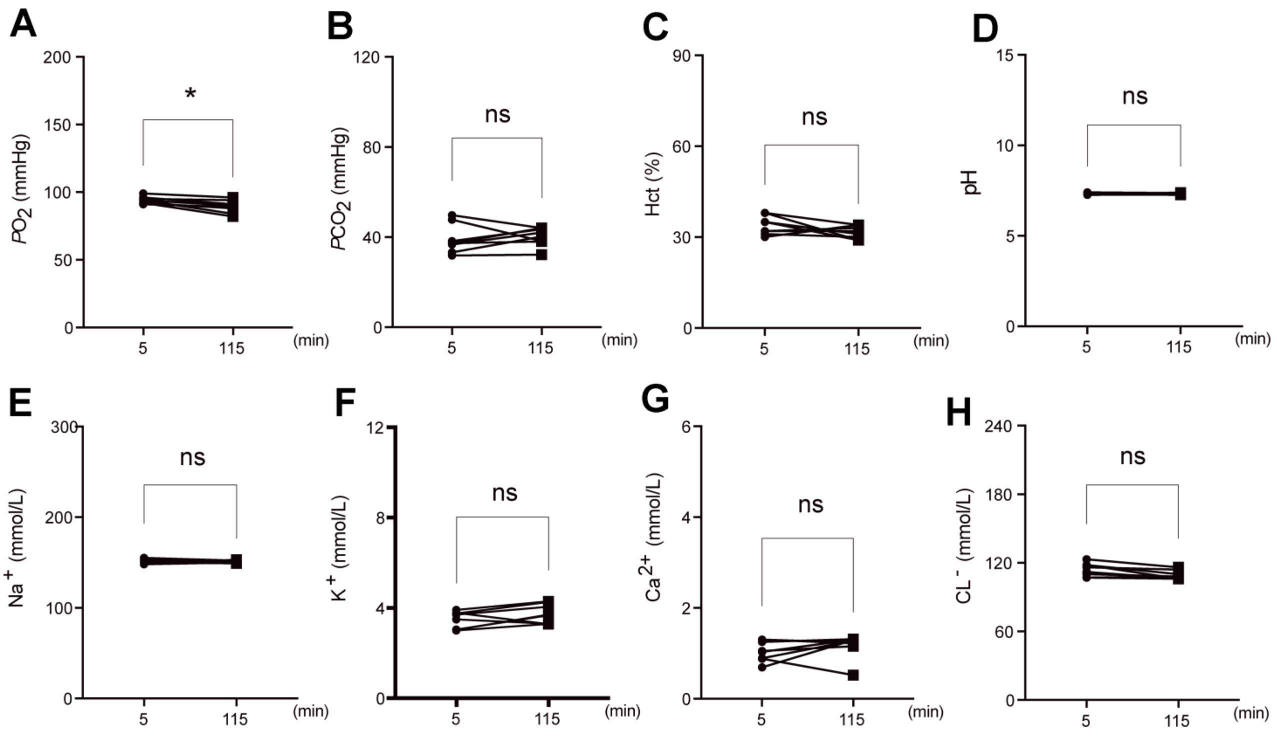

Supplementary Figure 2. Arterial blood gas analysis of mice receiving sevoflurane. Blood samples were taken at 5 min and 115 min during anesthesia on postnatal day 10. (**A**) PO2. (**B**) PCO2. (**C**) Hct. (**D**) pH. (**E**) Na+. (**F**) K+. (**G**) Ca2+. (**H**) Cl-. PO2, arterial oxygen tension; PCO2, arterial carbon dioxide tension; Hct, hematocrit. n = 8. *p < 0.05 for the comparison shown.

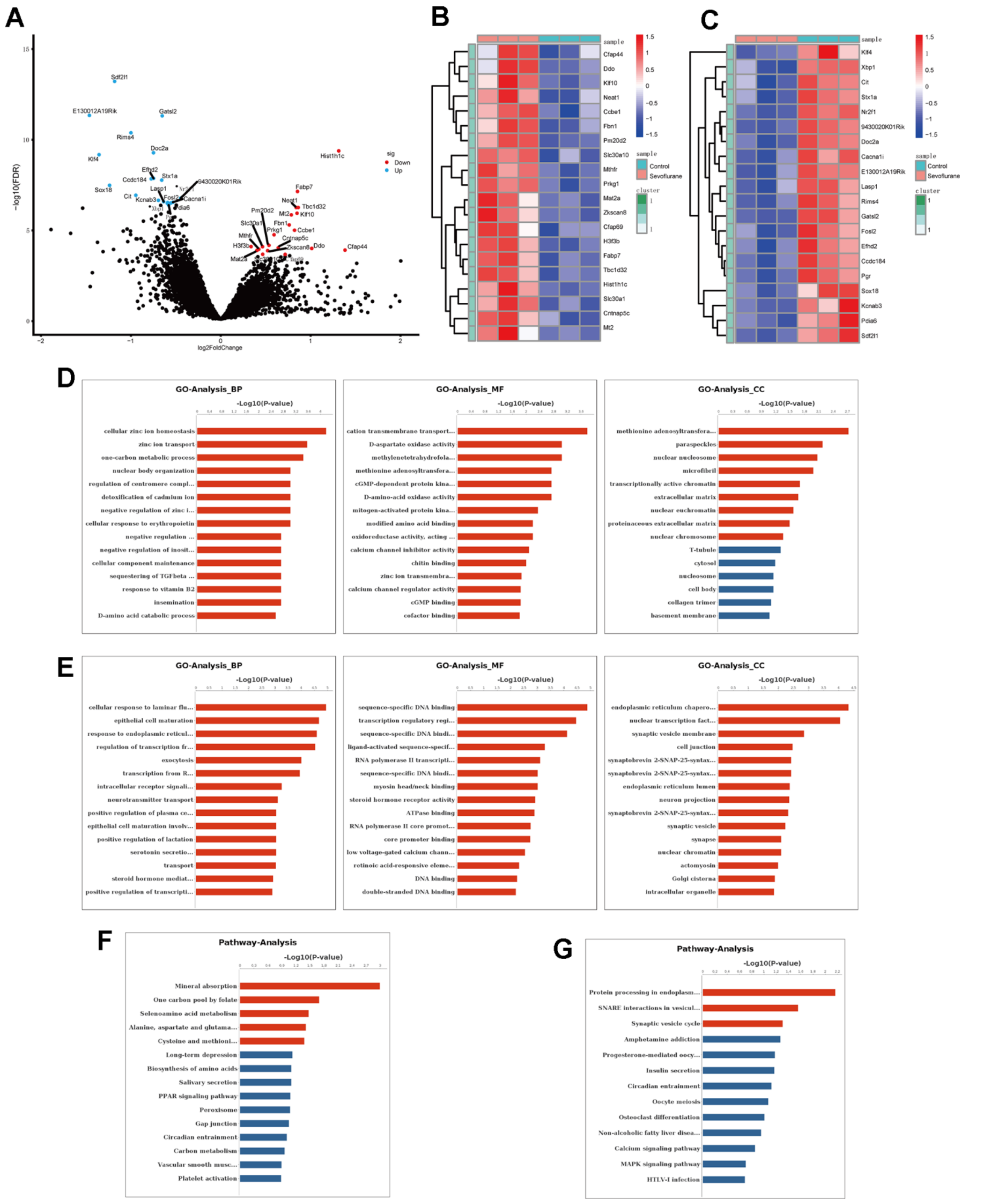

Supplementary Figure 3. Analysis of top 20 up- and down-regulated DEGs. (**A**) Top 20 up- and down-regulated DEGs labeled in volcano plot. (**B**) Heatmap of top 20 up-regulated DEGs. (**C**) Heatmap of top 20 down-regulated DEGs. (**D**) GO terms of BP, MF, and CC based on the top 20 up-regulated DEGs. (**E**) GO terms of BP, MF, and CC based on the top 20 down-regulated DEGs. (**F**) Pathway analysis of top 20 up-regulated DEGs. (**G**) Pathway analysis of top 20 down-regulated DEGs. BP, biological process; MF, molecular function; CC, cellular component. Blue columns indicate terms or pathways without significant difference.

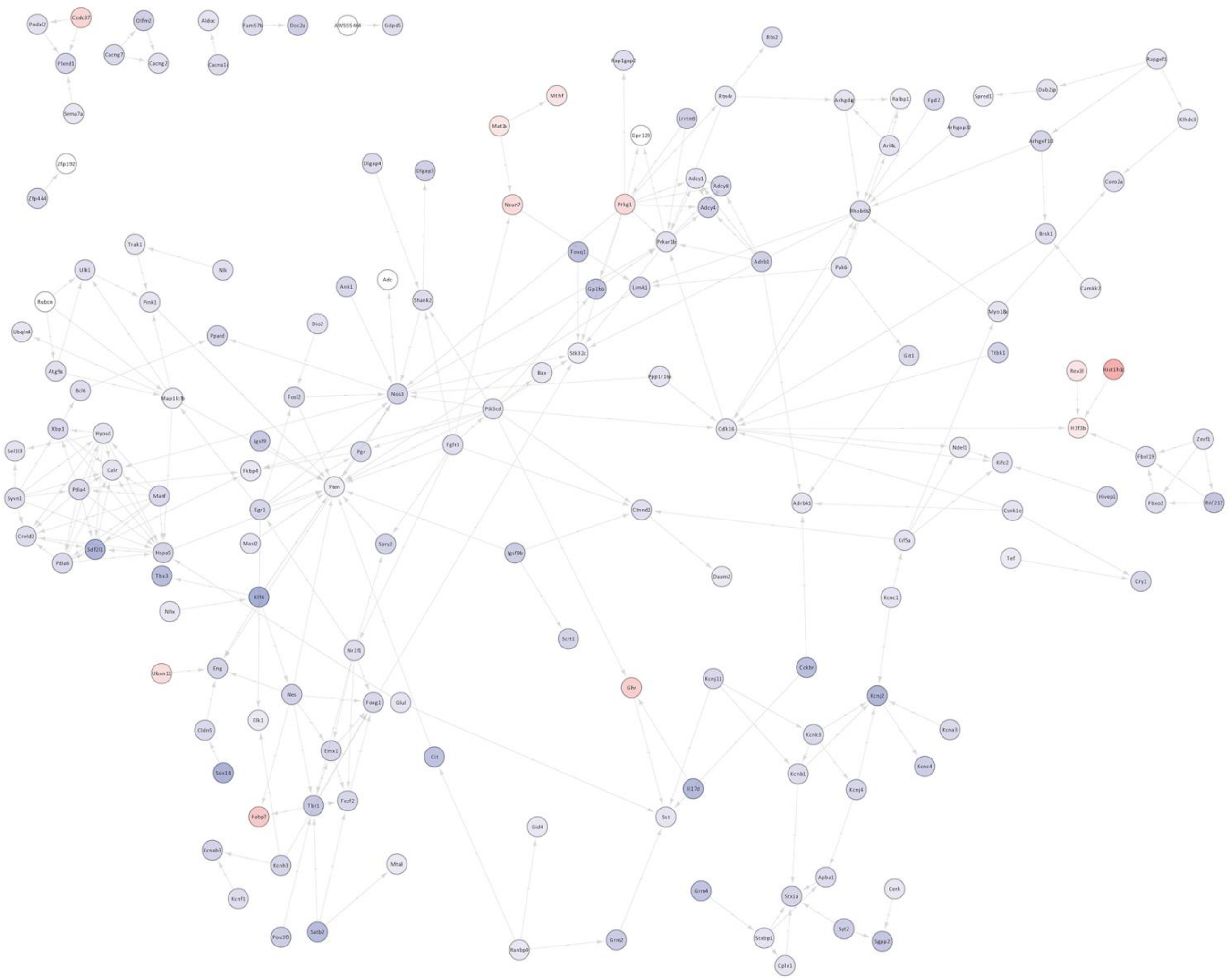

Supplementary Figure 4. PPI network of DEGs in hippocampal tissues between sevoflurane-treated and control mice.
