# Supplementary material for: Cognitive impairment and transcriptomic profile in hippocampus of young mice after multiple neonatal exposures to sevoflurane
Source: Aging (Albany NY). 2019 Oct 3;11(19):8386–417. doi: 10.18632/aging.102326 (PMC6814607; doi:10.18632/aging.102326)
Supplement: Supplementary Tables 1, 2, 10, 11 and 13 [file aging-11-102326-s009.pdf]

SUPPLEMENTARY TABLES

Supplementary Table 1. Primers specific to the top 20 up- and down-regulated DEGs.

Gene	Sequence (5′-3′)	Gene	Sequence (5′-3′)
Up-regulated		Down-regulated	
Hist1h1c-F	AAACTCAACAAGAAGGCGGC	Sdf2l1-F	GCTGCACTCACACGACATCA
Hist1h1c-R	CTTTAGACGCGCTCTTGACC	Sdf2l1-1R	TCCTGGTTGTTGGATAGCGG
Fabp7-F	TGATCCGGACACAATGCACA	E130012A19Rik-1F	TCCTCAGGACTCTCCGACC
Fabp7-R	CCATCCAACCGAACCACAGA	E130012A19Rik-1R	GCGGTTGAAATGTTCCTGGC
Neat1-F	CCTGCTGTCTGCTGGCACTTG	Gatsl2-F	AGACCCCTGAGGACTACACC
Neat1-R	CGTCTCCATCAACTGCTGCTCTG	Gatsl2-R	ACACGGAGATGTTCTGGTCG
Tbc1d32-F	TGCTTCTCACCGAAAACCCAT	Rims4-F	GATGAGGACGCAGCAGACAG
Tbc1d32-R	AAGCAAGCTCATTTTCTGCACC	Rims4-R	CTCCTGCAAGCCAATCTCCA
Klf10-F	CTCAACTTCGGCGCTTCTCT	Doc2a-F	ATGAGGACAAGCTGAGCCAC
Klf10-R	CTCTGCTCAGCTTTGTCCCA	Doc2a-R	CCGCAGACATTGAAGAGGGT
Mt2-F	CGCCTGCAAATGCAAACAATG	Klf4-F	TACCCCTACACTGAGTCCCG
Mt2-R	TCGGAAGCCTCTTTGCAGAT	Klf4-R	GGAAAGGAGGGTAGTTGGGC
Fbn1-F	GTGCAACTGGAAACGGGAAC	Efhd2-F	ACCCCTACACCGAGTTCAAG
Fbn1-R	GCGGGCAAATGCATCTGTAG	Efhd2-R	TCAGGTCGATGAAGCCATCC
Ccbe1-F	ACACGTGGACCTACCGAGAG	Ccdc184-F	ACGGCGGTATGAAGGAACTG
Ccbe1-R	CGCCCGAAGACTTCAGACAT	Ccdc184-R	ACAGAACCTGGATGTGCGAG
Prkg1-F	GGACGCAGAAGCAAAAGCAA	Stx1a-1F	ACCCCGATGAGAGCATTGAG
Prkg1-R	ACTCGTCCGAAACCTCCAAC	Stx1a-1R	TTTGCAGCGTTCTCGGTAGT
Pm20d2-F	CCGAGCACGATGTGACTGT	Sox18-1F	CCTTCTTCCCACCGCCTTT
Pm20d2-R	GGGCGCACGGAAGTAATAGA	Sox18-1R	GGTGGTGACAGAGGATTGGG
H3f3b-F	AAGAAGCCTCACCGCTACAG	Nr2f1-F	AGCGTCCGCAGGAACTTAAC
H3f3b-R	GTGACTCTCTTGGCGTGGAT	Nr2f1-R	TTCTTCCTCGCTGAACCGC
Slc30a1-F	TGGAAGCGGAAGACAACAGG	Cit-F	TTCCTGACGGGGACGTATCT
Slc30a1-R	AGACCAAGGCATTCACGACC	Cit-R	GTCATCGCCCTCCAGTTTCA
Cntnap5c-F	AGATTTACTCAGGCAATGTCACCT	9430020K01Rik-F	GAAGGGGGAGTCCACAACAG
Cntnap5c-R	GGGGGTACCAGTCAACAGC	9430020K01Rik-R	TCTAGTTCCCTGGAGAGCCC
Ddo isoform 1-F	ACAGTGTGTATTGCGGTCGT	Kcnab3-F	ATGCAGGTGTCTATCGCGTG
Ddo isoform 1-R	GTACCGGGGTATCTGCACAC	Kcnab3-R	CCAGGTACCAAGGCCAAGAC
Mthfr isoform a F	CCAGCTGGGCACTGTTATCC	Lasp1-F	CCCAAGCAGTCCTTCACCAT
Mthfr isoform a R	AGTCCATTCTGCGCCTCATC	Lasp1-R	CCACCACGCTGAAACCTTTG
Cfap44-F	TAAGAGCAGCAGCAAGCAGT	Cacna1i-F	GGCTGGACAGCGTCTCTTTA
Cfap44-R	CATAATCTCCAGCTCCGCGT	Cacna1i-R	CTCACCCTTGCTCTCCTTGG
Mat2a-F	AGCTGCTTATGCTGCTCGTT	Fosl2-F	CGGGAACTTTGACACCTCGT
Mat2a-R	TCAGATCCAGATCCCTGACAA	Fosl2-R	GGTGATGGCGTTGATTGTGG
Zkscan8-F	CACTGCAGGGTTCCAGACTT	Pdia6-F	CGTACTGCCTCACATCCTGG
Zkscan8-R	TGGCTGGATTCCGGGAGATA	Pdia6-R	GGTACCCGAACCCTCCAATC
Cfap69-F	GGATGGGGAGATGCAGGGTA	Xbp1-F	CTGAGTCCGCAGCAGGTG
Cfap69-R	GGTTCTACAAAACAGGGCTGC	Xbp1-R	AGGGAGGCTGGTAAGGAACT
Scl30a10-F	CTGCTCTCGGACTCGTTCAA	Pgr1-F	ATCTGGCTGTCACTATGGCG
Scl30a10-R	GCCTCCACGAAGATGGTGAA	Pgr1-R	TGCCAGCCTGACAACACTTT

Supplementary Table 2. All up-regulated DEGs.

AccID	log2FC	Pvalue	Style	AX3HiP1	AX3HiP6	AX3HiP7	C1HiP	C2HiP	C3HiP
Hist1h1c	1.31267581	5.32E-10	up	210	312	310	117	104	93
Fabp7	0.85361146	9.19E-08	up	510	585	626	287	348	263
Neat1	0.83695367	6.96E-07	up	2066	2703	3017	1280	1266	1470
Tbc1d32	0.86251088	7.10E-07	up	334	349	397	176	215	168
Klf10	0.84394199	1.46E-06	up	355	541	655	292	258	246
Mt2	0.79661629	1.74E-06	up	1721	2239	1968	1144	1201	913
Fbn1	0.75822236	6.25E-06	up	347	473	629	261	262	261
Ccbe1	0.81554074	1.23E-05	up	547	680	968	428	309	398
Prkg1	0.59114627	2.20E-05	up	351	358	508	279	248	223
Pm20d2	0.53770463	9.04E-05	up	334	399	545	283	289	242
H3f3b	0.3420804	0.00010199	up	3285	3577	4474	2977	2899	2479
Slc30a1	0.45620874	0.00010528	up	659	805	1034	589	632	473
Cntnap5c	0.63716696	0.00011373	up	335	377	429	277	222	192
Ddo	1.02095372	0.00013425	up	106	193	259	84	83	82
Mthfr	0.41618347	0.00014103	up	879	847	1227	754	695	608
Cfap44	1.38749371	0.00016006	up	233	474	629	91	136	218
Mat2a	0.40471043	0.00017422	up	3246	3280	3948	2675	2721	2074
Zkscan8	0.52475543	0.00017509	up	575	553	673	427	427	328
Cfap69	0.72324972	0.00026348	up	349	370	376	187	241	199
Slc30a10	0.47424661	0.00028382	up	475	495	709	372	436	315
Nphp3	0.67949682	0.00029212	up	294	270	318	181	203	139
Ghr	0.76690226	0.00032131	up	156	214	288	119	111	123
Zfp110	0.71562975	0.0003606	up	239	315	373	164	166	189
Rev3l	0.41260455	0.00036542	up	1385	1376	1726	1142	1045	969
Ccdc37	0.66879606	0.00038	up	168	184	270	115	129	116
Nsun7	0.55639805	0.00045858	up	308	307	381	226	241	171
Traf3ip2	0.70853818	0.00048953	up	295	242	292	181	169	133
Ubxn11	0.53699635	0.00052979	up	257	327	431	224	225	197
Lactb2	0.68114467	0.00059208	up	240	243	257	146	153	137
Col6a4	2.00241304	0.00062403	up	34	188	251	46	14	42
Slc22a4	0.65031318	0.00063502	up	150	160	230	119	116	85
Dsp	0.49510796	0.00064136	up	580	692	946	602	460	399
Adamts3	0.40837859	0.0006738	up	548	564	813	465	458	418
Gm15446	0.89449855	0.00068508	up	177	154	152	86	82	80
A430105I19Rik	0.55366746	0.00071236	up	216	221	306	162	164	144
Vwa3a	0.96074599	0.00079566	up	209	400	422	149	132	198
Zdhhc20	0.3640428	0.00080315	up	1019	1053	1309	893	852	718
Zfp941	0.60698307	0.00080663	up	346	448	495	230	305	253
Xlr3b	1.51243311	0.00084254	up	205	114	107	27	76	44
Kctd4	0.45136281	0.00091241	up	1069	1161	1235	846	833	716
Col25a1	0.54664464	0.0009775	up	1535	1889	3386	1367	1475	1341
Vprbp	0.37143641	0.00100464	up	689	757	935	632	605	487
Dcc	0.48492321	0.00101956	up	881	747	1284	769	655	509
Shtn1	0.4176569	0.00112328	up	732	812	1026	643	567	576
Tex9	0.73468468	0.00115775	up	131	206	214	109	103	96
Zkscan16	0.47956129	0.00116363	up	920	778	1004	679	645	511
A730017C20Rik	0.43986246	0.00122696	up	769	842	1029	620	737	476
Dbi	0.53083746	0.00125356	up	1778	2588	2876	1467	1537	1610
Glb1l	0.60406251	0.00127143	up	278	237	300	175	189	143

Please follow the links to see the data of Supplementary Tables 3–9.

Supplementary Table 3. All down-regulated DEGs.

Supplementary Table 4. All GO terms of biological process associated with up-regulated DEGs.

Supplementary Table 5. All GO terms of biological process associated with down-regulated DEGs.

Supplementary Table 6. All GO terms of cellular component associated with up-regulated DEGs.

Supplementary Table 7. All GO terms of cellular component associated with down-regulated DEGs.

Supplementary Table 8. All GO terms of molecular function associated with up-regulated DEGs.

Supplementary Table 9. All GO terms of molecular function associated with down-regulated DEGs.

Supplementary Table 10. All involved GO terms of biological process in GO-tree analysis.

GOID	GOTerm	Style	2GOID	2GOTerm	2Style	Relation
GO:0000122	negative regulation of transcription from RNA polymerase II promoter	down	GO:0006357	regulation of transcription from RNA polymerase II promoter	down	is_a
GO:0000122	negative regulation of transcription from RNA polymerase II promoter	down	GO:0006366	transcription from RNA polymerase II promoter	down	negatively_ regulates
GO:0000422	mitochondrion degradation	down	GO:0006914	autophagy	down	is_a
GO:0006171	cAMP biosynthetic process	down	GO:0009190	cyclic nucleotide biosynthetic process	down	is_a
GO:0006355	regulation of transcription, DNA-templated	down	GO:0006351	transcription, DNA-templated	down	regulates
GO:0006357	regulation of transcription from RNA polymerase II promoter	down	GO:0006355	regulation of transcription, DNA-templated	down	is_a
GO:0006357	regulation of transcription from RNA polymerase II promoter	down	GO:0006366	transcription from RNA polymerase II promoter	down	regulates
GO:0006366	transcription from RNA polymerase II promoter	down	GO:0006351	transcription, DNA-templated	down	is_a
GO:0006468	protein phosphorylation	down	GO:0016310	phosphorylation	down	is_a
GO:0006811	ion transport	down	GO:0006810	transport	down	is_a
GO:0006836	neurotransmitter transport	down	GO:0006810	transport	down	is_a
GO:0007269	neurotransmitter secretion	down	GO:0006836	neurotransmitter transport	down	is_a
GO:0007416	synapse assembly	down	GO:0007399	nervous system development	down	part_of
GO:0010107	potassium ion import	down	GO:0006813	potassium ion transport	down	is_a
GO:0010506	regulation of autophagy	down	GO:0006914	autophagy	down	regulates
GO:0014047	glutamate secretion	down	GO:0007269	neurotransmitter secretion	down	is_a
GO:0016358	dendrite development	down	GO:0031175	neuron projection development	down	part_of

GO:0018105	peptidyl-serine phosphorylation	down	GO:0006468	protein phosphorylation	down	is_a
GO:0018107	peptidyl-threonine phosphorylation	down	GO:0006468	protein phosphorylation	down	is_a
GO:0033563	dorsal/ventral axon guidance	up	GO:0007411	axon guidance	down	is_a
GO:0035212	cell competition in a multicellular organism	up	GO:0007275	multicellular organismal development	down	part_of
GO:0042977	activation of JAK2 kinase activity	up	GO:0042976	activation of Janus kinase activity	up	is_a
GO:0045893	positive regulation of transcription, DNA-templated	down	GO:0006351	transcription, DNA-templated	down	positively_ regulates
GO:0045893	positive regulation of transcription, DNA-templated	down	GO:0006355	regulation of transcription, DNA-templated	down	is_a
GO:0046929	negative regulation of neurotransmitter secretion	up	GO:0007269	neurotransmitter secretion	down	negatively_ regulates
GO:0048662	negative regulation of smooth muscle cell proliferation	down	GO:0008285	negative regulation of cell proliferation	down	is_a
GO:0050680	negative regulation of epithelial cell proliferation	down	GO:0008285	negative regulation of cell proliferation	down	is_a
GO:0060074	synapse maturation	down	GO:0007399	nervous system development	down	part_of

Supplementary Table 11. All KEGG pathways associated with up-regulated DEGs.

PathwayID	PathwayTerm	DifGene	AllDifGene	GeneInPathway	AllGene	P-Value	FDR	Enrichment	(-log10P)
PATH:04978	Mineral absorption	2	15	46	7456	0.003715	0.126321	21.61159	2.430003
PATH:03320	PPAR signaling pathway	2	15	81	7456	0.011168	0.189854	12.27325	1.95203
PATH:00670	One carbon pool by folate	1	15	19	7456	0.037585	0.333358	26.1614	1.424991
PATH:00450	Selenoamino acid metabolism	1	15	32	7456	0.062536	0.333358	15.53333	1.203869
PATH:00250	Alanine, aspartate and glutamate metabolism	1	15	37	7456	0.071971	0.333358	13.43423	1.142842
PATH:00270	Cysteine and methionine metabolism	1	15	40	7456	0.077589	0.333358	12.42667	1.110198
PATH:03460	Fanconi anemia pathway	1	15	51	7456	0.09792	0.333358	9.746405	1.009131
PATH:05210	Colorectal cancer	1	15	64	7456	0.121407	0.333358	7.766667	0.915757
PATH:04730	Long-term depression	1	15	73	7456	0.137331	0.333358	6.809132	0.86223
PATH:05412	Arrhythmogenic right ventricular cardiomyopathy	1	15	74	7456	0.139084	0.333358	6.717117	0.856723

	(ARVC)								
PATH:04970	Salivary secretion	1	15	77	7456	0.144322	0.333358	6.455411	0.840667
PATH:01230	Biosynthesis of amino acids	1	15	77	7456	0.144322	0.333358	6.455411	0.840667
PATH:04151	PI3K-Akt signaling pathway	2	15	346	7456	0.15169	0.333358	2.873218	0.819042
PATH:04146	Peroxisome	1	15	82	7456	0.152986	0.333358	6.061789	0.815347
PATH:04540	Gap junction	1	15	87	7456	0.161569	0.333358	5.71341	0.791643
PATH:04974	Protein digestion and absorption	1	15	89	7456	0.164979	0.333358	5.585019	0.782572
PATH:04512	ECM-receptor interaction	1	15	90	7456	0.166679	0.333358	5.522963	0.778119
PATH:04713	Circadian entrainment	1	15	97	7456	0.17849	0.337148	5.124399	0.748386
PATH:01200	Carbon metabolism	1	15	109	7456	0.198375	0.350059	4.560245	0.702513
PATH:04270	Vascular smooth muscle contraction	1	15	128	7456	0.228943	0.350059	3.883333	0.640273
PATH:04611	Platelet activation	1	15	130	7456	0.232097	0.350059	3.82359	0.634331
PATH:04722	Neurotrophin signaling pathway	1	15	133	7456	0.236805	0.350059	3.737343	0.62561
PATH:04360	Axon guidance	1	15	133	7456	0.236805	0.350059	3.737343	0.62561
PATH:05322	Systemic lupus erythematosus	1	15	144	7456	0.253837	0.359603	3.451852	0.595445
PATH:04630	Jak-STAT signaling pathway	1	15	155	7456	0.270514	0.3679	3.206882	0.56781
PATH:04022	cGMP-PKG signaling pathway	1	15	170	7456	0.292696	0.377713	2.923922	0.533583
PATH:05202	Transcriptional misregulation in cancer	1	15	175	7456	0.299949	0.377713	2.840381	0.522953
PATH:05034	Alcoholism	1	15	195	7456	0.328272	0.398616	2.54906	0.483766
PATH:04510	Focal adhesion	1	15	207	7456	0.344748	0.404188	2.401288	0.462498
PATH:04060	Cytokine-cytokine receptor interaction	1	15	260	7456	0.413109	0.466947	1.911795	0.383935
PATH:01100	Metabolic pathways	3	15	1188	7456	0.436412	0.466947	1.255219	0.360103
PATH:04080	Neuroactive ligand-receptor interaction	1	15	282	7456	0.43948	0.466947	1.762648	0.357061
PATH:05200	Pathways in cancer	1	15	325	7456	0.48786	0.502643	1.529436	0.311705
PATH:04740	Olfactory transduction	1	15	1067	7456	0.901626	0.901626	0.465854	0.044974

Please follow the kink to see the data of Supplementary Table 12.

Supplementary Table 12. All KEGG pathways associated with down-regulated DEGs.

Supplementary Table 13. All involved KEGG pathways in pathway network analysis.

PathID	PathTerm	Style	2PathID	2PathTerm	2Style
PATH:04015	Rap1 signaling pathway	down	PATH:04020	Calcium signaling pathway	down
PATH:04114	Oocyte meiosis	down	PATH:04914	Progesterone-mediated oocyte maturation	down
PATH:04141	Protein processing in endoplasmic reticulum	down	PATH:05020	Prion diseases	down
PATH:04261	Adrenergic signaling in cardiomyocytes	down	PATH:04020	Calcium signaling pathway	down
PATH:04540	Gap junction	down	PATH:04020	Calcium signaling pathway	down
PATH:04710	Circadian rhythm	down	PATH:04713	Circadian entrainment	down
PATH:04713	Circadian entrainment	down	PATH:04710	Circadian rhythm	down
PATH:04721	Synaptic vesicle cycle	down	PATH:04724	Glutamatergic synapse	down
PATH:04721	Synaptic vesicle cycle	down	PATH:04725	Cholinergic synapse	down
PATH:04721	Synaptic vesicle cycle	down	PATH:04727	GABAergic synapse	down
PATH:04723	Retrograde endocannabinoid signaling	down	PATH:04721	Synaptic vesicle cycle	down
PATH:04723	Retrograde endocannabinoid signaling	down	PATH:04020	Calcium signaling pathway	down
PATH:04723	Retrograde endocannabinoid signaling	down	PATH:04724	Glutamatergic synapse	down
PATH:04723	Retrograde endocannabinoid signaling	down	PATH:04727	GABAergic synapse	down
PATH:04724	Glutamatergic synapse	down	PATH:04721	Synaptic vesicle cycle	down
PATH:04724	Glutamatergic synapse	down	PATH:04020	Calcium signaling pathway	down
PATH:04725	Cholinergic synapse	down	PATH:04721	Synaptic vesicle cycle	down
PATH:04725	Cholinergic synapse	down	PATH:04020	Calcium signaling pathway	down
PATH:04727	GABAergic synapse	down	PATH:04721	Synaptic vesicle cycle	down
PATH:04742	Taste transduction	down	PATH:04020	Calcium signaling pathway	down
PATH:04911	Insulin secretion	down	PATH:05211	Renal cell carcinoma	down
PATH:04911	Insulin secretion	down	PATH:04020	Calcium signaling pathway	down
PATH:04912	GnRH signaling pathway	down	PATH:04020	Calcium signaling pathway	down
PATH:04913	Ovarian steroidogenesis	down	PATH:04912	GnRH signaling pathway	down
PATH:04915	Estrogen signaling pathway	down	PATH:04020	Calcium signaling pathway	down
PATH:04915	Estrogen signaling pathway	down	PATH:04913	Ovarian steroidogenesis	down
PATH:04915	Estrogen signaling pathway	down	PATH:04727	GABAergic synapse	down
PATH:04918	Thyroid hormone synthesis	down	PATH:04020	Calcium signaling pathway	down
PATH:04970	Salivary secretion	down	PATH:04020	Calcium signaling pathway	down
PATH:04971	Gastric acid secretion	down	PATH:04020	Calcium signaling pathway	down
PATH:05032	Morphine addiction	down	PATH:04727	GABAergic synapse	down
PATH:05166	HTLV-I infection	down	PATH:04115	p53 signaling pathway	down
PATH:05213	Endometrial cancer	down	PATH:04115	p53 signaling pathway	down
PATH:05221	Acute myeloid leukemia	down	PATH:04150	mTOR signaling pathway	down
